# Antiviral nanomedicine: Advantages, mechanisms and advanced therapies

**DOI:** 10.1016/j.bioactmat.2025.05.030

**Published:** 2025-06-05

**Authors:** Yicheng Pu, Chuanda Zhu, Jun Liao, Lidong Gong, Yijuan Wu, Shunquan Liu, Hongjun Wang, Qiang Zhang, Zhiqiang Lin

**Affiliations:** a Institute of Systems Biomedicine, Beijing Key Laboratory of Tumor Systems Biology, School of Basic Medical Sciences, Peking University, Beijing 100191, China; b Department of Biophysics, Peking University Health Science Center, Beijing 100191, China; c Department of Pharmaceutics, School of Pharmaceutical Sciences, Peking University, Beijing 100191, China; d School of Physics, Peking University, Beijing 100091, China; e Beijing Tide Pharmaceutical Co., Ltd, Beijing 100176, China

**Keywords:** Antiviral nanomedicine, Nanotechnology, Virus-host interaction, Targeted drug delivery, Biomimetic nanomaterials

## Abstract

The emergence of novel viral pathogens and the limitations of conventional antiviral therapies necessitate innovative strategies to combat persistent and pandemic threats. This review details the role of viral infections and antiviral nanomedicines, delving into the mechanisms of action and antiviral advantages of nanomedicines, as well as the latest research advances in this field. The review systematically categorizes the mechanisms of antiviral nanodrugs into a framework that integrates previously fragmented knowledge, and innovatively summarizes the unique attributes and advantages of antiviral nanodrugs compared to small-molecule drugs. Nanotherapies are proposed in this review to conclude advanced nanoantivirals (e.g., light-activated nanophotosensitizers, biomimetic decoys, PROTAC-based degraders, and gene-silencing platforms) and offer a distinctive narrative perspective, with the aim of presenting a merged and integrated overview of nanodrugs. By intuitively highlighting their commonalities in mechanisms or similarities in application methods, readers may better appreciate the innovative characteristics of different antivirals. We further discuss translational challenges and propose interdisciplinary solutions and future directions to accelerate the development of next-generation antiviral strategies. This review aims to inspire transformative research at the nexus of virology, nanotechnology, and precision medicine.

## Highlights

• This review systematically categorizes the mechanisms of antiviral nanodrugs into an innovative framework that integrates previously fragmented knowledge.
• Not limited to the general advantages of nanodrugs, this review innovatively summarizes the main attributes of antiviral nanodrugs, analyzing the unique advantages of nanomedicines and why they are particularly suited to antivirals.
• This review offers a discussion of the translational potential, implications for clinical applications and major directions for the future development of antiviral nanomedicines.

## Graphical abstract

**Figure:**
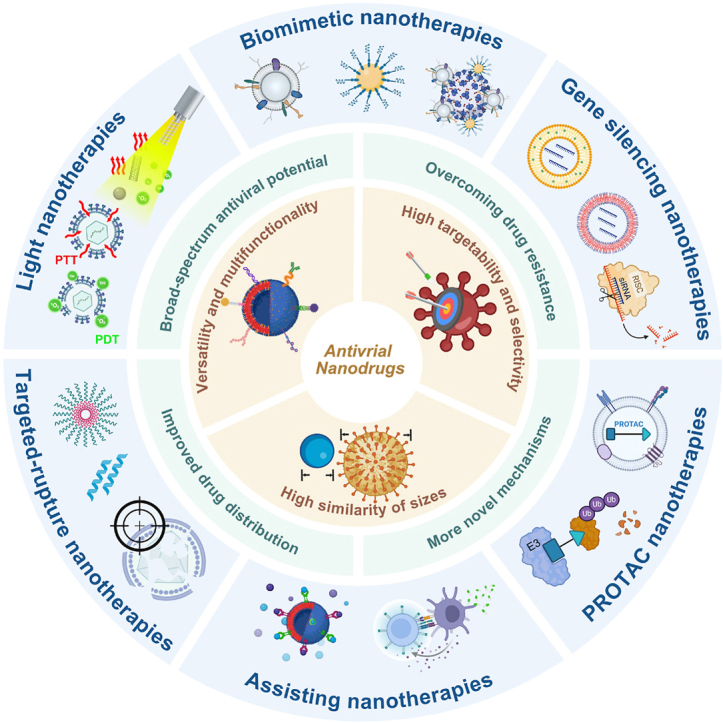

## 1 Introduction

The rise of globalization leads to frequent outbreaks and epidemics of viral diseases that threaten human health. Subsequent to the World Health Organization's declaration of the outbreak of the novel Coronavirus as a global pandemic on March 11, 2020, a total of 366 million confirmed cases and 5.6 million related fatalities have been reported on a global scale within a period of less than two years [1]. The emergence of new cases and mutant strains continues to be reported to the present day. By the end of 2023, the estimated global number for individuals infected with the Human Immunodeficiency Virus (HIV) was 39.9 million. More than 600,000 people are dying from HIV-related causes, with the number of people acquiring new HIV infections increasing annually [2,3]. In the era of globalization, the high risk of transmission and the rapid spread of viral diseases, such as HIV, influenza, and SARS-CoV-2, not only affect individual health but also have significant socio-economic consequences [4,5]. The development of preventive and therapeutic antiviral drugs and strategies is therefore necessary.

Vaccination is a common method of preventing viral infections. Nevertheless, the range of effective vaccines is presently extremely restricted. And for certain viruses characterized by high mutation rates, the efficacy of vaccines is known to wane in the emergence of novel strains [6,7]. Furthermore, despite the abundant resources of natural-derived antiviral drugs, they have not become the preferred choice in clinical antiviral therapy due to constraints such as complex active components, unclear mechanisms of action, purification challenges, and significant difficulties in industrial-scale production[[8], [9], [10]]. Since the approval of the first antiviral drug, idoxuridine, in June 1963, the use of chemically synthesized drugs has become the dominant antiviral treatment strategy of choice [11]. However, small molecule drugs suffer from significant limitations, such as limited efficacy, serious side effects, and resistance that arises from prolonged use[[12], [13], [14], [15]]. Currently, the process of developing new antiviral drugs is generally long, and there are corresponding clinically approved antiviral drugs for only a dozen of more than 220 viruses to which are known to be infectious to human [1,16]. Consequently, the necessity for the development of new antiviral strategies and pharmaceutical agents is becoming increasingly pressing.

In recent years, nanomedicine has become a subject of considerable research interest due to its biocompatibility and distinctive physical&chemical properties, which conferred by their nanostructures, including researches focused on antivirals [17,18]. Specific nanoscale drug carriers have the potential to enhance drug solubility and bioavailability, effectively reducing side effects[13,[19], [20], [21], [22], [23], [24], [25]]. Some nanobiomaterials can inactivate viruses and demonstrate novel antiviral mechanisms[[26], [27], [28]]. An increasing number of reports describe how nanodrugs can be successfully applied to block viral spread and deal with viral infection. The accelerated advancement of nanomedicine has opened new avenues for antiviral therapy.

In this review, we will critically analyze different therapies for the application of nanoparticles (NPs) as antiviral agents. For the sake of clarity, this review is broadly structured into four parts: an introduction to viral background knowledge, an analysis of the nano-antiviral mechanisms and advantages of nanodrugs, a narration of advanced antiviral nanotherapies, and a summary and outlook of current antiviral nanodrugs. In the following text, we first present background knowledge of viruses, including their classification and characteristics, as well as their mechanisms of infection and the body's immune response. Then, we innovatively summarize the mechanism of action and unique attributes of antiviral nanodrugs. Unlike the general advantages of NPs mention in prior reviews, we inductively analyzed the unique advantages of nanomedicines for antiviral therapy, summarized why they are particularly suitable for antivirals compared to small molecule drugs. It is noteworthy that authors of prior antiviral reviews mainly categorize nanodrugs by their material properties [7,29] or mechanisms of action [1,30] for narration. In this review we innovatively propose nanotherapies to summarize and conclude these different advanced nanodrugs. We expect to present a merged and integrated overview of nanodrugs which employ similar therapeutic strategies, intuitively highlighting their commonalities in mechanisms or similarities in application methods, while also listing their differences. By analyzing different nanodrugs under the same therapy, we aim to help readers to better appreciate the innovative characteristics of different medicines, thus stimulating new ideas for research on antiviral nanomedicine development.

## 2 Characterization and classification of the virus

The basic structure of a complete virus particle (virion) consists of nucleic acid (DNA or RNA) and a protein layer (capsid) to protect the nucleic acid, and some viruses also form an envelope outside the capsid [31,32]. Viruses are unable to survive independently and require the host cell to invade and utilize its mechanisms for replication [33]. The range and variety of viruses are vast, with more than 320,000 mammal-infecting viruses on Earth, according to scientific studies and modelling calculation [34].

**Fig. 1:**
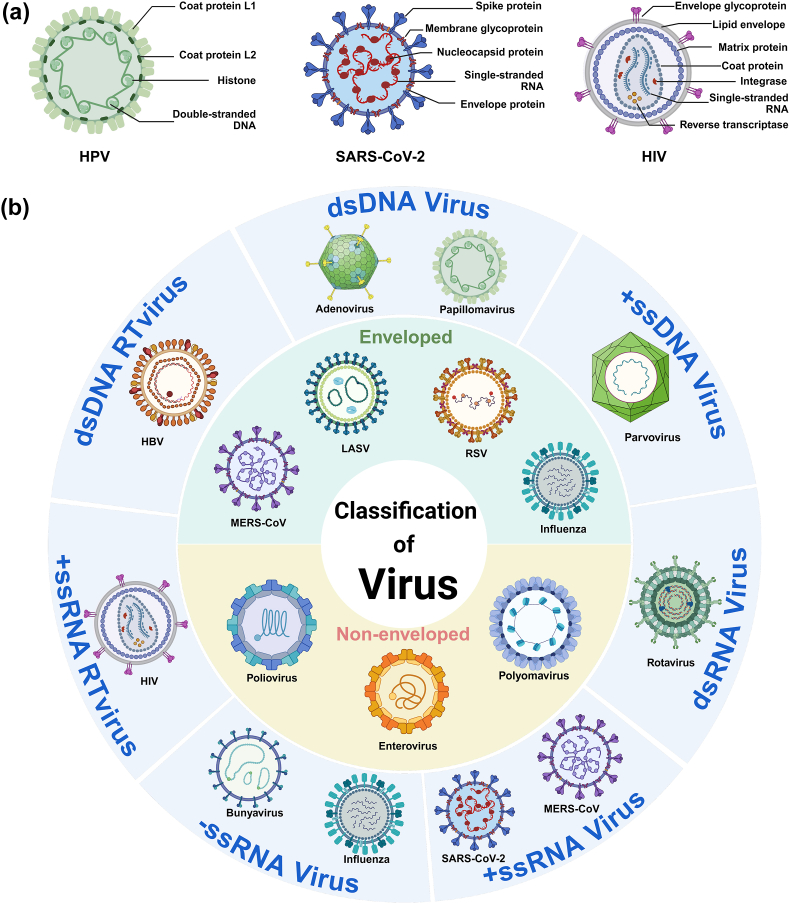
a) Schematic diagram of the structures of several prevalent enveloped/non-enveloped viruses b) Classification based on whether the virus has an envelope and the Baltimore Classification System. Created in https://BioRender.com.

With regard to the viruses that have been identified and subjected to scientific research, scientists have classified them in accordance with their different structural and genetic characteristics. The classification of viruses can be divided into two categories, namely enveloped and non-enveloped, depending on whether the virus is enveloped or not [35]. Enveloped viruses possess a lipid membrane that originates from the host cell membrane and surrounds the protein coat. The lipid membrane plays an indispensable role in maintaining viral integrity and stability, protecting viral genetic material, and achieving viral replication. Membrane surface glycoproteins, such as haemagglutinin and neuraminidase, serve to identify and bind to receptor sites on host cell membranes to enter the cell [36]. Examples of such viruses include HIV and coronaviruses (Fig. 1a) [[37], [38], [39]]. In contrast, non-enveloped viruses lack a lipid membrane and the genetic material is typically protected by capsid [40]. The capsid consists of protein subunits that assemble into highly symmetrical shapes through non-covalent bonds like hydrogen bonds and hydrophobic interactions. The dense structure of the capsid confers non-enveloped viruses greater environmental resistance, examples of such viruses include the human papilloma virus (HPV) and poliovirus [41,42].

Besides the basic structures of nucleic acids and capsids, some virus particles also have their own transcription-related enzymes, like RNA-dependent RNA polymerase (RdRp) found in influenza viruses and reverse transcriptase present in HIV[[43], [44], [45]]. That is because viruses contain various types of genetic material, and the ways they convert this genetic information into mRNAs recognized by host cells differ. Virologist David Baltimore developed the Baltimore Classification System [[46], [47], [48]], which categorizes viruses into seven main categories based on their genome type and replication strategy [49,50] (Fig. 1b):double-stranded DNA virus (dsDNA virus), single-stranded DNA virus (+ssDNA virus), double-stranded RNA virus (dsRNA virus), positive single-stranded RNA virus (+ssRNA virus), negative single-stranded RNA virus (-ssRNA virus), single-stranded RNA retrovirus (+ssRNA RTvirus) and double-stranded DNA retrovirus (±dsDNA RTvirus).

## 3 Mechanism of infection and how the body responds

**Fig. 2:**
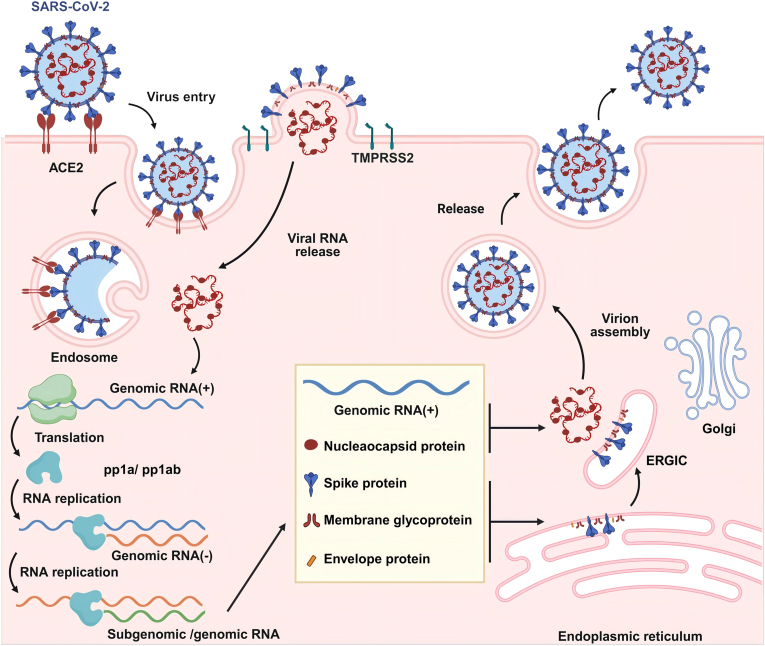
Schematic diagram of the infection lifecycle of SARS-CoV-2 as the representative virus. Created in https://BioRender.com.

The replication cycle of viruses comprises six principal steps, namely attachment, entry into the cell, uncoating, replication and transcription-translation of the genome, assembly and release [51,52]. The first step in infection is the attachment of viral particles to susceptible cells. The virus attaches to specific receptors on cell surfaces using capsid or envelope proteins. With the help of certain lipids or glycoproteins, it can then enter the host cell through either membrane fusion or endocytosis [53]. After entry, the virus migrates to a specific replication site, where it then begins uncoating and replication. This migration process post-internalization is known as intracellular trafficking, and microtubule assisted transportation along with receptor-mediated endocytosis is the major intracellular transport mechanism for viruses [54,55]. Uncoating is the process by which a virus releases its genetic material from its protein coat, which is a prerequisite for its subsequent replication and transcription. Afterward, the virus synthesizes nucleic acids and proteins using nucleotides and other materials from the host cell [56]. Most enzymes needed during this period originate from the host cell, while some viruses carry essential replication enzymes, including RdRp in SARS-CoV-2 [57] and reverse transcriptase in HIV [58]. Following the synthesis of viral nucleic acids and proteins, they are assembled in the nucleus or cytoplasm to form nucleocapsid. The mature nucleocapsids of non-enveloped viruses are released from the cell through lysis. Some enveloped viruses complete their assembly by acquiring an envelope through the budding of the nucleocapsid [59]. While the assembly of other enveloped viruses occurs in the endoplasmic reticulum (ER)/Golgi intermediate compartment (ERGIC), where the packaged virions are transferred to the cell surface and released via exocytosis to infect other cells [[60], [61], [62]] (Fig. 2).

Upon infection by a virus, an organism's immune protection mechanisms are activated, encompassing both innate and adaptive immunity [63]. Innate immune cells recognize the presence of viruses by engaging in multiple ligand-receptor interactions. Specifically, pattern recognition receptors (PRRs), such as Toll-like receptors (TLRs) and RIG-I-like receptors (RLRs), recognize pathogen-associated molecular patterns (PAMPs). This recognition amplifies the signaling cascade involving various intracellular regulators, activates downstream signaling pathways, and ultimately leads to the synthesis and release of pro-inflammatory cytokines and interferons [64,65]. Pro-inflammatory cytokines recruit and activate immune cells and enhance local immune defenses [66], while interferon induces cells to produce a variety of antiviral proteins, such as protein kinase R (PKR) [67] and 2′-5′ oligoadenylate synthetase (OAS) [68], as well as inhibiting the proliferation of virus-infected cells [69]. Meanwhile, dendritic cells mature and engulf pathogens, processing them to present their antigenic fragments, which activate T and B cells to mount an adaptive immune response. The B-cell surface receptor (BCR) recognizes the antigen and transforms into a plasma cell, which produces antibodies that bind to the antigenic proteins, preventing the virus from attaching to target cell receptors, which in turn stops further infection [70]. When T lymphocytes are activated by an antigen, CD4^+^ and CD8^+^ T cells proliferate and differentiate in large numbers. CD8^+^ effector T cells act as killers by recognizing and removing infected cells, while CD4^+^ T cells enhance the function of CD8^+^ T cells and help B cells produce a stronger, longer-lasting antibody response [71]. Furthermore, some T and B cells are transformed into memory cells that can survive in the body for months or even years, allowing for a quick immune response when the body encounters the same virus again [72], which is why the vaccine plays a preventive role.

## 4 Mechanisms of antiviral nanodrugs

The current preferred strategy for antiviral therapy is to select small molecule drugs that interfere with the viral infection cycle—blocking one or more stages of the process [73]. Conventional antiviral drugs can be organized into the following six categories based on their mechanisms of action [11,32]: ①inhibition of viral nucleic acid synthesis: these include nucleoside analogues such as 5-substituted 2′-deoxyuridine analogues (Idoxuridine), acyclic guanosine analogues (Aciclovir), acyclic nucleoside phosphonate analogues (Adefovir Dipivoxil), as well as inhibitors of viral DNA/RNA synthesis-related enzymes such as pyrophosphate analogues (Foscarnet Sodium), nucleoside reverse transcriptase inhibitors (Zidovudine, Lamivudine) and non-nucleoside reverse transcriptase inhibitors (Nevirapine); ②prevention of viral precursor protein cleavage: protease inhibitors (Lopinavir, Boceprevir); ③inhibition of integration of viral DNA into the human chromosomes: integrase inhibitors (Raltegravir); ④viral entry inhibitors: Maraviroc; ⑤inhibition of viral release from infected cells: influenza virus neuraminidase inhibitors (Oseltamivir, Zanamivir); ⑥immunostimulant: interferon inducer (Imiquimod), or direct administration Interferon.

**Fig. 3:**
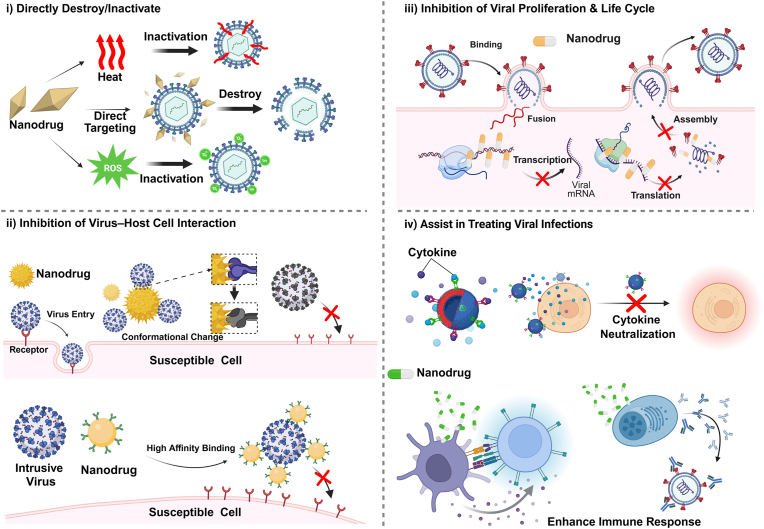
Schematic of possible action mechanisms of antiviral nanodrugs. Nanodrugs can i) directly destroy/inactivate viruses, ii) inhibit virus–cell interaction to inhibit viral infection, iii) disrupt viral proliferation and viral life cycle, iv) assist in treating viral infections. Created in https://BioRender.com.

However, nanotechnology provides a number of unique mechanism of nanomedicines over conventional antiviral drugs with regard to viral suppression [74,75]. Here, we summarized the mechanism of action of some existing nano-antiviral drugs, schematically shown in Fig. 3.

①**Direct destruction & inactivation of virus.** Some NPs oxidize and destroy envelopes or proteins in the viral capsid by releasing metal ions or generating free radicals [30,76], for example, CuxO-TiO_2_, which will be mentioned later in the context of light nanotherapy. There are also some novel nanomaterials that, due to their unique physicochemical properties, induce viral structural damage through hydrogen bonding, electrostatic interactions and redox reactions activated by thermal reduction to achieve antiviral effects [1],e.g. negatively charged graphene oxide nanosheets with sharp edges have increased chances of interacting with positively charged virus particles through electrostatic interactions, leading to virus destruction and inactivation [77].
②**Inhibition of virus-host cell interactions.** Viruses are devoid of a complete enzyme system, the necessary raw materials and energy to synthesize their own components, and ribosomes. This determines their parasitic nature and the need to infect the host cell to complete the production of offspring [53]. Some nanomedicines are designed to bind with high affinity to viruses and block virus-cell interactions by competing for virus-host cell binding sites[[78], [79], [80]]. For example, silver nanoparticles interact with the HIV-1 virus via preferential binding to the gp120 glycoprotein knobs, inhibiting the virus from binding to host cells [81]. Some nanomedicines, on the other hand, interfere with the recognition of cellular receptors by altering the structure of interacting proteins on the surface of the virus [82]. For example, Neal et al. [83] employed Ag to modified cerium oxide nanoparticles with 53.7 % Ce(III), which oxidatively denatured receptor-binding proteins in through the large number of oxygen vacancies on their surface and the presence of Ag^+^. The antiviral effect is achieved by inhibiting this interaction and preventing the virus from parasitizing within the host cell.
③**Inhibition of viral proliferation & life cycle after infection.** Following the interaction and invasion of the host cell, viruses exhibit parasitic properties and replicate themselves within the intracellular environment. Some nanomedicines hinder viral genome expression by binding to viral DNA (or RNA) and inhibiting transcription and translation processes [84,85]; Or disrupt the normal replication cycle by targeting enzymes and proteins critical to viral replication [86]. For example, Mashino et al. [87] functionalized nanofulleren with anionic and cationic pyrrolidinium salts as well as amino acids and found its inhibition for HIV reverse transcriptase (HIV-RT). There are also some nanomedicines targeting infected host cells and triggering signaling pathways to regulate the intracellular environment and to inhibit the of virus-induced cell changes, thus preventing the spread of the virus. For example [88], VP1 siRNA functionalized silver NPs effectively increased the level of AKT, inhibited the activation of caspase-3 and restrained the expression levels of p53 in Enterovirus 71 (EV71) infected Vero cells. Such nanomedicines have multiple targets, but generally speaking, they seek to disrupt the normal replication of the virus and affect its life cycle through various pathways.
④**Assist in treating viral infections.** In addition to the administration of symptomatic treatments that are directed towards viruses, nanodrugs have the capacity to fulfill an adjunctive role in the treatment of infections. Some nanodrugs have been found to be capable of trapping cytokines, thereby reducing both local and systemic inflammation and calming cytokine storms [89,90], which may avoid some extent of tissue damage caused by viral infections and reduce the risk of complications due to inflammation. Some nanobodies with the capacity to neutralize viruses has been undertaken to a certain extent, thus providing a measure of assistance to humoral immunity in patients following viral infections [91]. Some nanodrugs possess immune-activating properties, such as NanoMn, which can stimulate immune cells and promoting interferon expression [92,93]. These properties can help restore immunity and repair defense mechanisms compromised by viral infections. They also enhance immune-antiviral functions and decrease the risk of secondary bacterial or other pathogenic infections.

Furthermore, some drugs act as cargos and are delivered with the utilization of nanomaterials or nanocarriers, exerting their own distinct antiviral effects. For instance, antiviral siRNA that function by selectively targeting viral genes and inhibit viral proliferation can be delivered via liposomes in antiviral gene-silencing therapy [94].Some mRNA that encoding viral antigens have the potential to be vaccines and delivered by nanocarriers [95,96]. In conclusion, nanocarriers perform diverse antiviral functions through different cargos, which enhances the efficacy of antiviral drugs by endowing them with the advantages of nanotechnology.

## 5 Why nano?

Despite significant development in antiviral drugs, there are numerous challenges associated with the currently approved antiviral drugs, including poor oral bioavailability and limited absorption of acyclovir, as well as reduced penetration of zidovudine due to its high water solubility [97,98]. In order to achieve sufficient blood concentration to exert a therapeutic effect, the common method is to increase the frequency of administration or the dose of the drug. However, this approach also presents certain challenges, including the potential for serious side effects, such as gastrointestinal tract reactions, hepatotoxicity, nephrotoxicity, cardiotoxicity, myelosuppression [32,97,99,100]. In conclusion, there is scope for enhancement and optimization of the existing conventional treatment for viral infections. However, the advent of nanotechnology has precipitated the development of nanomedicine, which has been a boon for antivirals. To enhance drug stability and solubility and improve bioavailability, one can either reduce particle size to the nanoscale or utilize nanocarriers, such as liposomes and polymers, for encapsulation [101,102]. It is noteworthy that these enhanced drug stability and improved bioavailability are general advantages of nanodrugs. In this section, the emphasis is placed on their unique advantages in the field of antivirals. These advantages can be attributed to several attributes of nanodrugs.

### 5.1 High similarity of sizes

Compared with conventional small molecule drugs, nanoparticles and viral particles have a high similarity of sizes (on the same order of magnitude) [103]. The distinctive properties of this nanomedicine render it especially suitable for interacting with viral particles, thereby achieving potent antiviral efficacy. Small molecule drugs exert their antiviral effects primarily through interactions with proteins or nucleic acids, thereby inhibiting the infection process. Nanomedicines with comparable particle sizes exhibit a range of unique antiviral properties that conventional small molecule drugs do not have, however. For example, as nanodecoys to trap viral particles [104], competitively binding to viral particles [30], occupying recognition sites with host cells and interacting with cell membranes to block viral penetration [27], etc. At the same time, due to the size similarity of NPs and viral particles, they may have similar in vivo kinetics, which makes it easier for the nanomedicine to interact with viral particles and exert therapeutic effect.

### 5.2 High targetability and selectivity

Small molecule drugs circulate throughout the body via the bloodstream, which exhibit relatively lower accumulation in infected host cells and tissues with high viral concentrations [105,106]. Nanomedicines possess abundant surface and internal structures, which can be subjected to various modifications. By conjugating specific targeting molecules to the surface of nanomedicines, active recognition and binding to specific cells or viral particles can be achieved, thereby enhancing targeting and selectivity. For example, nanodrugs with N-Acetylgalactosamine conjugation are liver-targeted and can be enriched in the liver to exert antiviral effects [107,108]. Meanwhile, cells infected and damaged by viruses exhibit differences from normal cells, such as the pH value of the cell surface [109]. Nanomedicines can be designed to be sensitive to these differences, thereby specifically accumulating in the lesion tissues or cells and exerting antiviral effects.

### 5.3 Versatility and multifunctionality

The versatility of antiviral nanodrugs is mainly reflected in the diversity of materials and the diversity of mechanisms of action. With the rapid development of nanomaterials, many biocompatible materials have been applied to the research of inhibiting viral infections, such as graphene oxide, carbon nanotubes, and nanometal particles[[110], [111], [112], [113]], which have greatly expanded the range of antiviral drugs. Furthermore, the antiviral diversity is reflected in multiple functionalities and mechanisms for inactivating viruses. This can be attributed to the unique physical & chemical properties of nanomaterials. Some nanomaterials can adsorb viral particles and even inactivate them through van der Waals forces [114] and electrostatic interactions [115]. Other nanomaterials possess redox activity, enabling them to react with proteins or nucleic acids on the viral surface [83], thereby damaging the structure and function of the virus. Notably, certain NPs also combine different mechanisms of antiviral action all in one, e.g. AgNPs can interact not only with cell membranes to block viral penetration, but also with viral genomes and viral factors necessary for viral replication [27,30]. NPs can also be modified and functionalized to combine multiple therapeutic effects, e.g. some engineered cell membrane-derived NPs can simultaneously neutralize both viruses and cytokines [89], demonstrating the unique advantages of nanomedicines in the antiviral field.

**Fig. 4:**
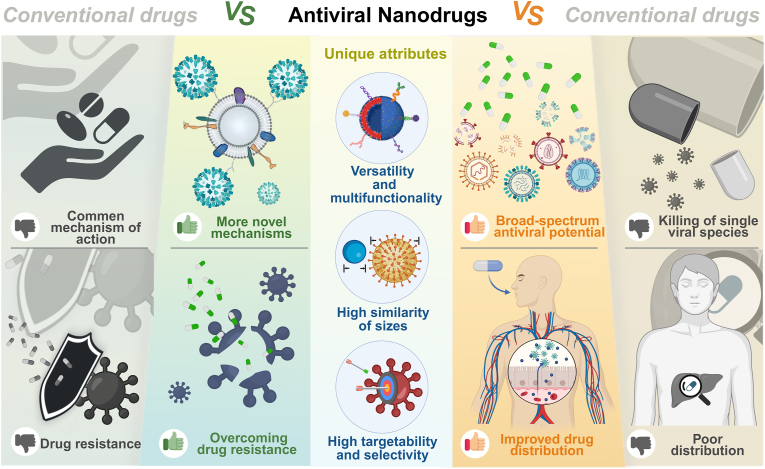
Limitations of conventional antiviral drugs and unique attributes and advantages of nanodrugs. Created in https://BioRender.com.

Therefore, on the basis of the above characteristics and attributes, our summary of the advantages of nanodrugs for the antiviral field is as follows (Fig. 4): ① More novel mechanisms of action: Nanomedicines provide antiviral therapy through mechanisms that are not available with small molecule drugs, such as nanodecoys to capture viral particles [116], nanoknife to mechanically destroy viral particles [77], generation of reactive oxygen species (ROS) to inactivate viral particles [117,118], and so on. ②Broad-spectrum antiviral potential: Small molecule drugs often have a single target of action, e.g. oseltamivir inhibits the neuraminidase enzyme that is specific to influenza viruses and is ineffective against other viruses [119]. While some nanodrugs destroy viruses through mechanisms such as van der Waals forces and electrostatic gravity, which have a broad spectrum of viral particle killing potential. ③Overcoming drug resistance from mutations: By powerfully disrupting the physical structure of viral particles as described above, particularly the non-viral genetically encoded viral lipid membrane (envelope), nanodrugs can avoid viral resistance that is often observed under the use of small-molecule drugs [120]. They provide a means of coping with drug-resistant viral strains as well. ④Improved drug distribution: Thanks to high targetability and selectivity and the high similarity of sizes, NPs are more likely to interact with viral particles to exert effects, while avoiding the impact on normal tissues and reducing side effects.

## 6 Advanced nanotherapy & nanodrugs

**Fig. 5:**
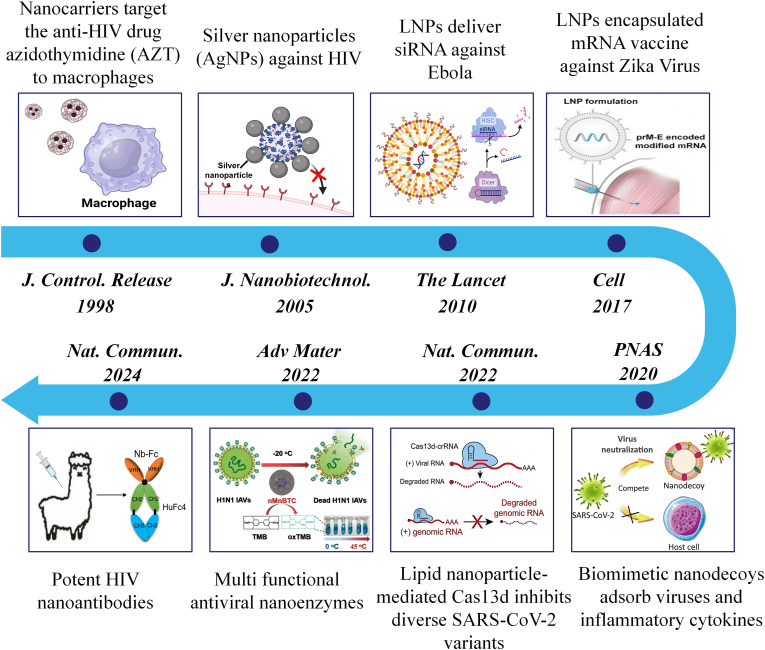
Schematic representation of milestones of antiviral nanodrugs[81,[121], [122], [123],[128], [129], [130]]. Reproduced with permission [123]. Copyright 2017, Elsevier. Reproduced with permission [122]. Copyright 2020, National Academy of Sciences. Reproduced with permission [130]. Copyright 2022, Springer Nature. Reproduced with permission [128]. Copyright 2022, Wiley-VCH. Reproduced with permission [129]. Copyright 2024, Springer Nature.

As nanotechnology advances, antiviral nanomedicines have evolved through various stages of development. Here, we present the timeline of antiviral nanomedicines' development and categorize it into three distinct phases (Fig. 5): ①nanocarrierization of conventional drugs, ②nanodelivery of novel drugs, and ③the phase of new technology/new mechanism in nanomedicine. In the early development of nanotechnology-enabled antiviral drugs, scientists focused on improving the deficiencies of existing small molecule drugs with nanotechnology, such as using colloidal drug nanocarriers to deliver azidothymidine (AZT), improving macrophage targeting and reducing toxicity [121]. The development of molecular biology has introduced novel drugs, including siRNA that silences virus-related genes [122] and mRNA that produces virus-related antigens [123]. These drugs, which are delivered using liposomes and other nanocarriers, provide a new idea of nanoantiviral therapy, and have also achieved clinical acceptance [124,125]. After the COVID-19 outbreak, there has been a significant rise in interest in antiviral drug discovery and development. Many materials scientists have entered this field, bringing new perspectives on antivirals. At this phase, research on new biotechnological drugs and materials for antiviral use is growing quickly, such as nanocleavers that use CRISPR/Cas9 to eliminate viral DNA [126,127] and nanoenzymes that have both antiviral and anti-inflammatory effects [128].

In this section, we focus on the advanced nanodrugs emerged in Phase ② and Phase ③, summarizing and categorizing them into different nanotherapies based on their therapeutic effect, the mechanism/target of their action and their own unique properties. We categorize and describe new antiviral nanotherapies according to the timeline of viral infection: killing and destroying viral particles before the viral invasion (light nanotherapies and targeted-rupture nanotherapies), interfering during the viral proliferation cycle (biomimetic nanotherapies, proteolysis-targeting chimeras (PROTAC) nanotherapies, and gene silencing nanotherapies), and the treatment after the onset of the viral-infected disease (assisting nanotherapies).

### 6.1 Light nanotherapy

**Fig. 6:**
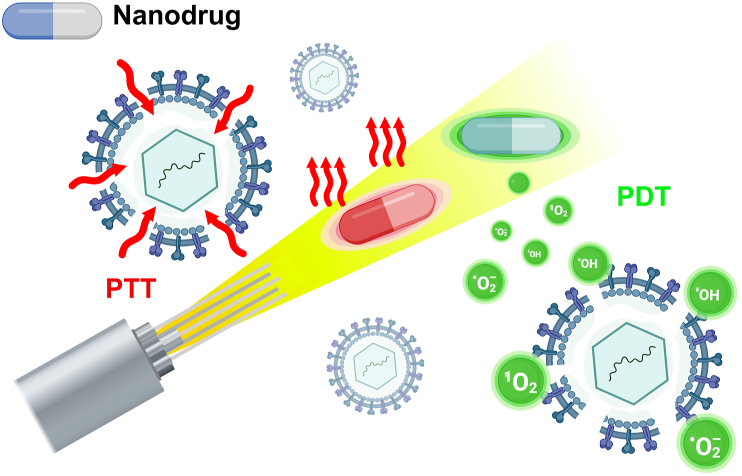
Schematic diagram of antiviral nanodrugs in light nanotherapy.

With the development of cutting-edge technologies such as nanotechnology, photonic and medical technologies, light is gradually being applied to solve problems in the medical field, especially in the treatment of diseases such as tumors and vascular lesions through methods such as photothermal therapy (PTT) and photodynamic therapy (PDT) [131,132]. Similarly, it has shown potential in certain infectious diseases, such as viral infections (Fig. 6).

**Fig. 7:**
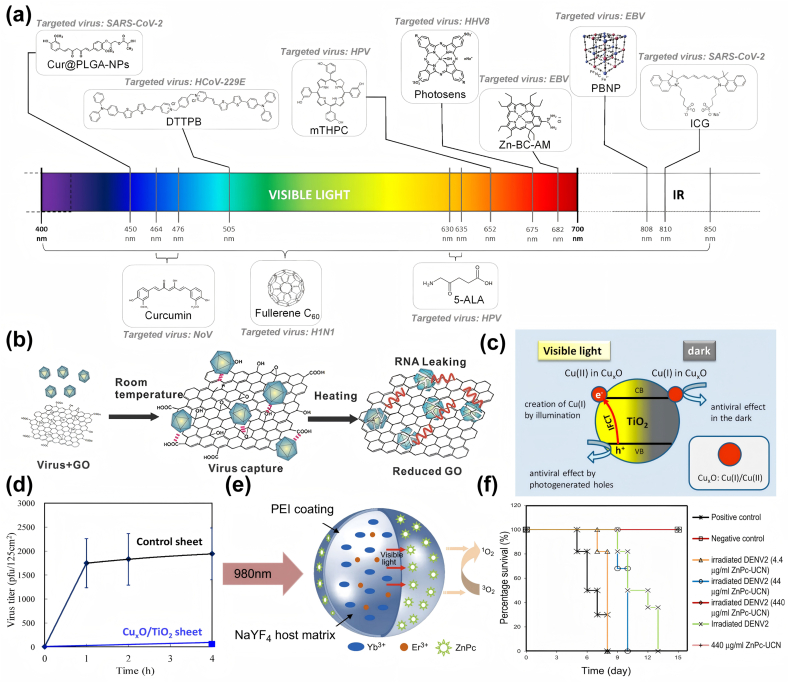
a) Photosensitizing molecules that exhibit antiviral activity under visible and/or infrared wavelengths in antiviral phototherapy. Reproduced with permission [164]. Copyright 2022, MDPI. b) Schematic illustration of the GO and virus interaction. Reproduced with permission [165]. Copyright 2014, WILEY‐VCH. c) Schematic illustration of the working principle of Cu_x_O/TiO2 photocatalyst in antiviral therapy. d) The Cu_x_O/TiO2-coated sheet produced a robust inactivation of bacteriophage Qβ in comparison to the control sheet without the photocatalyst. Reproduced with permission [157]. Copyright 2020, MDPI. e) Schematic drawing of UCN structure and mechanism of action of UCN-based PDT; f) Kaplan–Meier survival curve of BALB/c mice that were inoculated with photodynamically inactivated DENV2, showing the possibility to eradicate Dengue virus pathogenesis in BALB/c mice through ZnPc-UCNPs phototherapy. Reproduced with permission [162]. Copyright 2011, Elsevier.

PDT is a non-invasive treatment that involves the combination of a photosensitizer with specific wavelengths of light, thereby producing a therapeutic effect [133]. Photosensitizers are compounds that absorb certain wavelengths of electromagnetic radiation (UV, visible light, IR) and exhibit biological activity. Upon absorption of a light quantum, the photosensitizer forms singlet excited state (S_1_), which is subsequently converted to a triplet state (T1) by intersystem crossing (ISC). Triplet excited state is followed by ROS production via a type I photoreaction or a type II photoreaction and its own inactivation [134]. Type I photoreactions involve the transfer of electrons from T_1_ to the substrate or surrounding molecules such as oxygen; whereas in type II photoreactions T_1_ directly exchange energy with a triplet oxygen molecule (^3^O_2_) to produce singlet oxygen (^1^O_2_). Numerous types of ROS are produced by PDT, with the type I photoreaction producing superoxide anion (O_2_^−^·) intermediates, which can be subsequently disproportioned to H_2_O_2_ and catalyzed by metal ions to produce hydroxyl radicals (·OH)[[134], [135], [136]]. In addition, the generated ·OH and ^1^O_2_ initiate a lipid peroxidation chain reaction by attacking lipid molecules and combining with oxygen to form lipid peroxyl radicals (LOO·). Either way, generated ROS can cause damage to biomolecules [137]. Recent studies have demonstrated that the lipid envelope of enveloped viruses, which is abundant in unsaturated fatty acids, is highly susceptible to oxidation by ROS in comparison to non-enveloped viruses that exhibit greater resistance to ROS, and to cellular membranes that demonstrate a certain degree of resistance to oxidative stress[[138], [139], [140]]. It is the increased susceptibility to ROS exhibited by enveloped viruses that allows PDT to be used as a strategy for antiviral therapy (Fig. 7a) [141,142].

Photothermal agent (PTA) is a material or molecule that absorbs light energy at specific wavelengths and efficiently converts it into heat. Photothermal therapy is a therapeutic modality that uses a PTA to convert light energy into heat energy under near-infrared (NIR) light irradiation, which has been widely studied and uesd in cancer treatment [143]. Recently, however, antiviral PTT has been explored and developed. Nanoparticles with photothermal conversion capabilities or other PTA-modified nanoparticles are irradiated with a laser of short pulse duration and activated, which sharply raises the vibrational energy, leading to an increase in the local temperature of the tissue. When viral particles are exposed to such localized high temperatures, damage is caused to the viral structure (e.g. protein denaturation, nucleic acid damage) or key enzymes [144],which leads the inactivation of the virus.

In this subsection, we categorize antiviral nanodrugs that operate under light conditions into carbon-based and metal-based groups based on their material attributes, creating a narration of light nanotherapy.

#### 6.1.1 Carbon-based antiviral phototherapy

Carbon nanotubes (CNTs) are cylindrical hollow nanomaterials, which are microtubes composed of rolled-up sheets of graphene with planar sp2 hybridization [145]. The formation of multi-walled carbon nanotubes (MWNTs) by concentrically rolling up graphene layers serves to increase the surface area of CNTs and, by extension, the number of functionalization sites. The modification of CNTs with peptides, glycans or pharmaceuticals has been demonstrated to reduce their lung toxicity and high hydrophobicity, thus lowering the application limitations in the field of medication use [146]. In a study [118], Banerjee et al. functionalized and modified multi-walled carbon nanotubes with the photosensitizer protoporphyrin IX and explored their antiviral capabilities. Protoporphyrin IX (PPIX) is the final intermediate in the hemoglobin biosynthetic pathway and is an endogenous substance. It is extensively employed as a photosensitizer in photodynamic therapy due to its radiodensities properties and ROS production at specific wavelengths [147]. It was found that under conditions of visible light irradiation for 90 min, the infection rate of NCI cells by the influenza A virus (IAV) treated with PPIX-MWNT decreased from 78 % in the absence of irradiation to approximately 1 %. Furthermore, the inactivation of the virus by the PPIX-modified nanomaterials was found to be considerably enhanced in comparison to that of the unfunctionalized multi-walled nanotubes. Through exploring the mechanism of PPIX-MWNT inactivation, the researchers concluded that this inactivation of influenza A viruses under these conditions may be attributable to various factors, including the production of ROS leading to protein oxidation, the occurrence of single-strand breaks in the RNA genome, and the formation of protein-RNA cross-links. PPIX-modified multi-walled carbon nanotubes offer several advantages in the treatment of viral infectious diseases. One such advantage is that the carbon nanotubes act as scaffolds, thereby facilitating the recovery of photoreactive PPIX from solution. This property of MWNTs thus renders them a reproducible antiviral agent. In addition, since the generation of ROS under PDT can be lethal to a wide range of viruses, this PPIX-modified NT exhibits non-specificity to viruses and has the potential to be explored for the treatment of a wide range of viral infections.

Graphene nanomaterials, particularly graphene oxide (GO, Fig. 7b) and reduced graphene oxide (RGO), are widely explored in medical fields such as cancer photothermal therapy due to their distinctive property of efficiently absorbing light within the NIR spectrum [148]. In the domain of photothermal antiviral therapy, functionalized RGOs have demonstrated noteworthy strength. It has been shown that functionalized graphene nanomedicines (SMRGO) obtained by sulfonated magnetic nanoparticle modification of RGO sheets can significantly enhance their capture efficiency and photothermal properties for virus particles. Gedanken et al. [149]. conducted a comparative analysis of the capture ability and photothermal antiviral efficacy of magnetic nanoparticles (MNPs) and SMRGO against the herpes simplex virus type 1(HSV-1) virus. The study revealed that the recruitment capability of both materials to virus particles was comparable; however, under NIR irradiation, SMRGO exhibited a significant antiviral activity, with a 100 % killing rate of HSV-1, while MNPs demonstrated limited efficacy in this regard. SMRGO's effectiveness extends to a wide range of viral infections, including EV71, influenza virus H9N2, and other viral infections. It is important to highlight that the localized elevated temperatures in PTT not only damage viral particles but also have an impact on normal tissues and cells. It has been suggested that the use of longer-wavelength, lower-energy light improves the efficiency and accuracy of PTT while causing less damage to adjacent cells and tissues [150,151]. However, taking advantage of nanodrugs to recruit viral particles for PTT seems to further reduce this damage. SMRGO provides a solution idea. SMRGO combines the advantageous properties of graphene's larger surface area, special lamellar structure and excellent photothermal properties with the magnetic properties of MNPs and the advantages of recruiting viruses, which are able to polymerize captured viruses to a single point, further improving the efficacy of photothermal therapy, thus showing more effective and rapid antiviral effects.

#### 6.1.2 Metal-based antiviral phototherapy

In addition to the previously mentioned carbon-based nanophotosensitizers, antiviral metallic nanomaterials with phototherapeutic properties have attracted much attention in recent years. Among them, titanium oxides exhibited excellent photocatalytic properties and antiviral capabilities [152,153]. TiO_2_ has been demonstrated to possess the capacity to facilitate photo-oxidation, a process which can result in the inactivation of viruses such as Hepatitis B virus (HBV) and HSV. This capability arises from the generation of ROS (e.g. hydroxyl radicals and peroxides) upon light absorption through electron transfer reactions involving oxygen and water [154]. However, in contrast to conventional photosensitizers, TiO_2_ is not activated by visible light but by ultraviolet (UV) light. Consequently, researchers sought to shift the absorption spectrum of TiO_2_ towards the visible region by doping it with other elements [155]. They discovered that TiO_2_ doped with other metal elements significantly enhanced its photocatalytic inactivation of bacteria and viruses. Benjawan et al. [156] prepared Ag/TiO_2_ nanoparticles by the peroxo sol-gel method and achieved almost 100 % killing efficacy against H1N1 virus and EV 71. They note that the augmented photocatalytic virucidal activity of Ag/TiO_2_ in comparison with TiO_2_ can be ascribed to the elevated productivity of the actives (OH· and H_2_O_2_). Miyauchi et al. [157] modified TiO_2_ with Cu_x_O and verified its antiviral activity (Fig. 7c). Cu_x_O contains Cu(I) and Cu(II) valence states. Cu(I) catalyzes the generation of hydroxyl radicals via Fenton and Fenton-like reactions, which has shown to exert antiviral activity by oxidizing the capsid proteins and damaging viral components [30,158]. Under visible light irradiation, light-induced interfacial charge transfer, Cu(II) in Cu_x_O nanoclusters acts as an electron acceptor to form Cu(I) with antiviral activity, while photogenerated holes with strong oxidative capacity are formed in the TiO_2_ valence band, which enhances the inactivation ability of Cu_x_O-TiO_2_ (Fig. 7d). The advantages of photosensitive TiO_2_ nano-antiviral drugs are that they are relatively stable under photocatalysis, easy to produce and use, inexpensive, and pose no risk to the environment or human beings. Moreover, existing studies have shown that photoactivated TiO_2_ performs exceptionally well in inactivating various pathogens, including hepatitis C virus (HCV), H1N1 influenza virus, HSV-1, Zika virus, etc., demonstrating its broad-spectrum antiviral properties [1].

Furthermore, rare earth metal-related light-mediated antiviral nanotherapeutics have also been the focus of research interest. Rare earth elements are a group of elements in the third subgroup (3B) of the periodic table, which includes the lanthanide elements, as well as scandium (Sc) and yttrium (Y). Rare earth element atoms have a unique electronic configuration with partially filled 4f orbitals, which provides rich electronic energy levels and many electron transition channels, enabling a wide variety of radiation absorption and emission alongside unique optical properties [159,160]. Upconversion nanoparticles (UCNPs) are a class of lanthanide-doped nanocrystals that have the capacity to convert two or more low-energy photons into a single high-energy output photon (e.g., visible or UV) under near-infrared (NIR) excitation [161]. This property renders them a potential candidate for applications in PTT and PDT.

Lim et al. [162] synthesized UCNPs through the doping of Ytterbium (Yb^3+^) and Erbium (Er^3+^) ions with sodium yttrium fluoride (NaYF4) nanocrystals, which were then coated with high molecular weight polyethyleneimine (PEI) and modified with the photosensitizer molecule zinc phthalocyanine (ZnPc). When ZnPc-UCNPs were exposed to NIR light at 980 nm, UCN emitted visible light, which was absorbed by the photosensitizer ZnPc on the surface. Subsequent to activation, ZnPc converts nearby molecular oxygen into ROS, leading to viral killing (Fig. 7e). This study demonstrated the effectiveness of ZnPc-UCNPs in reducing the viral titers of two distinct viruses: the enveloped virus Dengue virus serotype 2 (DENV2) and the non-enveloped virus Adenovirus serotype 5 (Ad5V). In the case of HepG2 cells infected with DENV2, the release of the virus from infected cells during the recovery period (two days after treatment) was observed, suggesting that ZnPc-UCNPs play a positive role in the inactivation of the virus in infected cells. Furthermore, the researchers observed no in vivo pathogenicity of the light-treated DENV2 virus suspension when inoculated into mice, suggesting that the rare earth-doped nanophototherapeutic drug was able to effectively inactivate the virus and reduce the infectivity to a level that did not pose a threat to the mice (Fig. 7f). The combination of such photosensitizers with rare-earth optically characterized UCNPs allows the use of NIR, which has a better depth of penetration into living tissues compared to the direct use of photosensitizers absorbing visible light for phototherapy. This demonstrates the potential of phototherapeutic antiviral strategies in diseases such as thick lesions and localized infections of dermatological warts.

In the context of viral infections, the primary mechanism of action of light nanotherapy relies on the generation of ROS or thermal energy to kill the virus itself and/or destroy the infected host cells. However, extant research on these phototherapy-related nanomedicines has focused on isolated viruses or in vitro cellular models, and it is uncertain to what extent they can be used in humans for systemic viral infections, particularly in deep organ tissues, which is the focus of subsequent research. Nevertheless, antiviral nanophototherapy shows promise in targeting superficial localized viral infections and can also be used for sterilization of various biological or blood products. For example, Pourhajibagher et al. [163] reported the application of curcumin polylactic acid-hydroxyacetic acid nanoparticles (Cur@PLGA-NPs) for the inactivation of SARS-CoV-2 viruses in the plasma of contaminated patients under blue light. Therefore, further research and development of antiviral phototherapeutic nanomedicines is required in order to enhance the thermal conversion properties of photosensitizers and to achieve higher responsiveness to light at strong tissue-penetrating wavelengths. In addition, exploration of the possibility of targeting systemic viral infections in conjunction with other external-field stimuli (e.g. ultrasound) is also necessary.

### 6.2 Targeted-rupture nanotherapy: nanodrugs that act as snipers

In the domain of antiviral therapy, the optimal target for drug action is the viral particle itself, with the ideal efficacy being the elimination of only the viral particle. Consequently, researchers are exploring antiviral treatments that directly destroy viral particles with minimal impact on normal cells and tissues and that interfere with normal cells as little as possible. Nevertheless, this is a challenging task that demands meticulous attention to the differences between viral particles and normal cells. Only by using this difference as an antiviral ‘scope’ will we be able to ‘snipe’ viral particles more accurately and efficiently.

**Fig. 8:**
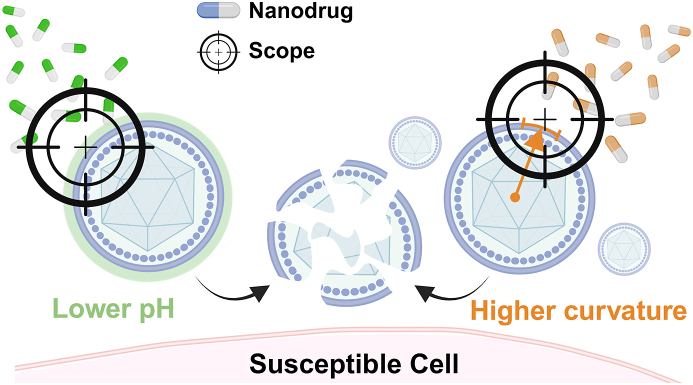
Schematic diagram of antiviral nanodrugs in targeted-rupture nanotherapy. Created in https://BioRender.com.

For enveloped viruses, the lipid membrane is a common feature and plays an indispensable role in maintaining viral integrity and stability, protecting viral genetic material, and achieving viral fusion and replication. The envelope has been employed by researchers as a target for the development of antiviral drugs and has demonstrated broad-spectrum anti-viral potential[109,[166], [167], [168]]. However, it is important to note that the lipid bilayers of enveloped viruses originate from host cells, which means selective targeting of viral envelopes rather than the membrane of healthy host cells necessitates a focus on what makes the viral envelope unique and distinctive. In this section, we summarized two unique nanotherapies that target ruptured viral envelopes (Fig. 8). They use the lower pH and higher curvature of the viral envelope as ‘scopes’ to rupture it for antiviral efficacy, respectively.

#### 6.2.1 Lower pH as ‘scope’

It has been established that in infected host cells, viruses induce alterations to the cell's lipid membrane through the incorporation of self-encoded proteins. These proteins include viral pore proteins [169]. This protein increases membrane permeability to hydrogen ions and is able to mediate the release of hydrogen ions from the Golgi to the extracellular compartment, thereby affecting the local pH of the cytoplasmic membrane. Therefore, given the provenance of the post-infection viral envelope from acidified host cell membranes, pH may also be considered a potential drug target for the viral envelope[39,109,[169], [170], [171]].

**Fig. 9:**
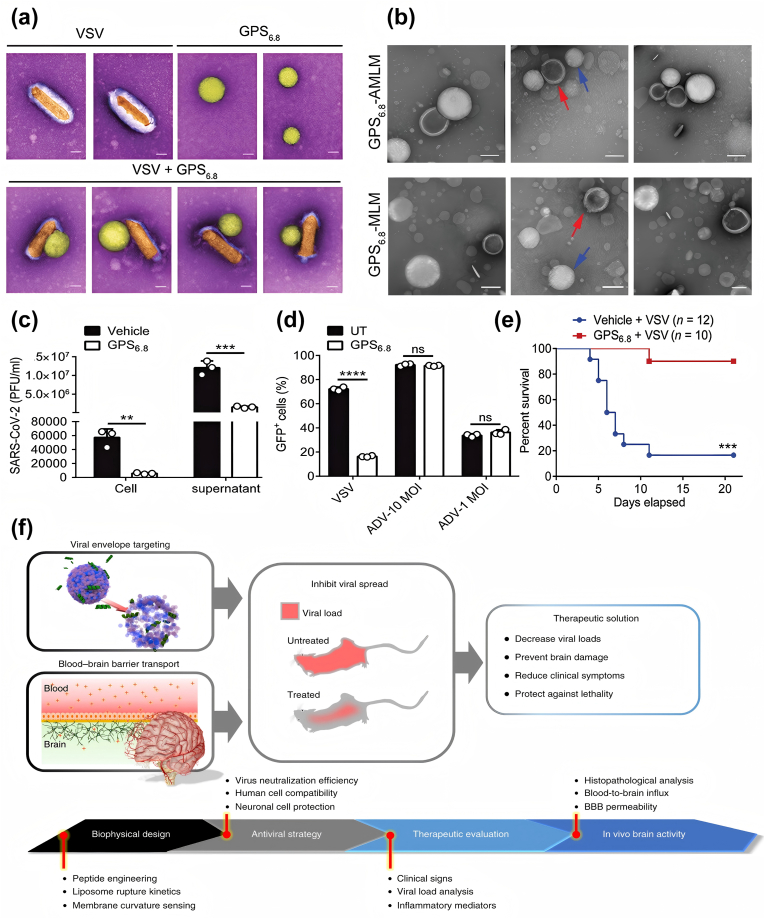
a) After co-incubation of GPS_6.8_ with VSV, disruption of the viral envelope was observed under TEM. Scale bars:100 nm. b) GPS_6.8_ selectively interacts with AMLM, causing rupture of its lipid membrane. Scale bars: 100 nm; (Red arrows indicate MLM or AMLM, while the blue arrows indicate GPS_6.8_) c). Titers of SARS-CoV-2 in NHBE cells infected with 10 MOI SARSCoV-2 in the presence or absence of GPS_6.8_ for 18 h were determined by qRT-PCR analysis. d) Flow cytometry analysis of HeLa cells infected with VSV-GFP (0.01 MOI) or indicated titers of ADV-mNeonGreen, and treated with GPS_6.8_ in the meantime. e) Survival rates were higher when treated with GPS_6.8_. Reproduced with permission [109]. Copyright 2022, Wiley‐VCH. f) Schematic illustration of LEAD strategy and development of antiviral brain-penetrating nanopeptides AH-D that inhibited Zika virus in an in vivo mouse model. Reproduced with permission [167]. Copyright 2018, Springer Nature.

Our team reported on a nanopolymer, GPS_6.8_, designed to disrupt the viral envelope [109]. GPS_6.8_ is a copolymer consisting of polyethylene glycol (PEG), 40 % of monomer of 2-(diethylamino)ethyl methacrylate (DEA) and 60 % of monomer of 2-(dipropylamino)ethyl methacrylate (DPA),which dissociates from nanoparticles into linear polymers at pH 6.8. In the construction of a gradient pH-sensitive (GPS) polymer nanoprobes library, we discovered that GPS_6.8_ nanoparticles selectively interact with the membranes of virus-infected cells. Moreover, to our delight, under Transmission Electron Microscopy (TEM), we observed that GPS_6.8_ directly adsorbed onto the surface of virus particle in vitro, disrupting the viral envelope (Fig. 9a). To investigate whether the low pH of the viral envelope could be targeted for direct virus destruction, we designed acidified liposomes (AMLM) encapsulating fluorescent dyes to mimic the viral envelope and incubated them with GPS_6.8_. TEM images showed that GPS_6.8_ selectively interacted with AMLM, and significant membrane damage of AMLM was also observed, demonstrating that GPS_6.8_ indeed induced rupture of the viral membrane (Fig. 9b). Fluorescence changes in the AMLM and in the non-acidified liposome MLM before and after administration were observed by means of confocal microscopy. The results showed that GPS_6.8_ significantly reduced the fluorescence intensity of AMLM, indicating lipid membrane rupture and fluorescent dye leakage, whereas there was no significant change in the fluorescence signals of MLM, successfully demonstrating that lower pH can indeed be employed as a target for antiviral drugs that directly disrupt the viral envelope.

Meanwhile, the GPS_6.8_ has shown potent activity against a wide range of viruses in vitro, limiting the spread of not only vesicular stomatitis viruses (VSV), but also RNA viruses such as SARS-CoV-2 (Fig. 9c) and lymphocytic choroid plexus meningitis virus (LCMV), and DNA viruses such as HSV. However, this inactivation property is effective only for enveloped viruses and does not work on non-enveloped viruses, such as Adenovirus (Fig. 9d), because GPS_6.8_ targets the acidified envelope of the virus. In a VSV-infected C57BL/6 mouse model, the treatment group injected with GPS_6.8_ via the tail vein showed lower mortality (Fig. 9e), attenuated lung pathological changes and lower viral titers in the liver, lung and spleen compared to the control group (vehicle injection only). This suggests that GPS_6.8_ may be beneficial in reducing mortality from viral infections, inhibiting in vivo replication and transmission of viruses, and attenuating inflammatory lung damage. These results provide an experimental basis for a potential antiviral therapeutic tool such as pH-sensitive nanodrugs targeting the viral envelope, and demonstrate the feasibility of using pH as a target for disrupting the viral envelope.

#### 6.2.2 Higher curvature membranes as ‘scope’

The enveloped viruses exhibit smaller particle sizes and higher lipid membrane curvature in comparison to host cells [172]. In response to the difference in curvature between the viral envelope and the membranes of mammalian cells, Cho et al. proposed a lipid envelope antiviral disruption (LEAD) strategy and developed antiviral nanopeptides that preferentially target high-curvature lipid membranes (Fig. 9f) [167]. They selected an amphipathic α-helical (AH) peptide containing 27 amino acids as a template. This structure was found to interact with the phospholipid bilayer of the viral envelope; and the AH peptide was engineered with d-amino acids, which are more resistant to protein hydrolysis, with a view to improving the stability and potency of the peptide. A size-selective and potent antiviral peptide, AH-D, was finally prepared. In vitro experiments demonstrated that the AH-D peptide quickly disrupts the liposomes of simulated virus particles. It suggests that the peptide compromises viral structures and inhibits replication by either damaging the lipid envelope or creating pores, which ultimately reduces infectivity. In virus neutralization assays, the AH-D peptide demonstrated potent neutralizing activity against the Zika virus (ZIKV) with a diameter of approximately 50 nm, while it did not inhibit larger-sized enveloped or non-enveloped viruses (e.g., the smallpox virus and poliovirus). In the ZIKV-infected mouse model, mice treated with AH-D peptide had significantly improved survival and significantly lower viral loads in serum and spleen compared to the untreated group, where all mice died. The results presented above illustrate the feasibility of targeting the lipid membrane curvature of enveloped viruses. This suggests that broad-spectrum antiviral nanopeptides targeting the viral envelope are a viable proposition, and provides a new idea for the future development of broad-spectrum antiviral drugs.

Targeted-rupture antiviral nanotherapy that directly destruct viral particles has a number of advantages. Firstly, lipid membranes serve as a common structure for enveloped viruses, which means this nanotherapy has a broad spectrum of inactivation of a wide range of enveloped viruses. Secondly, taking targeted-rupture nanotherapy as an entry point of antiviral research can avoid viruses from evading the drug action through mutation, since lipid biosynthesis is not encoded in the viral genome [173]. Moreover, although the preceding section describes the use of PDT to generate ROS, which can also disrupt the viral envelope, ROS production inevitably affects normal cell membranes. As outlined in this section, the utilization of unique physical structure or basic chemical properties of virus particles as 'scopes', such as the low pH and higher curvature of the envelope, renders this therapy highly selective for viral particles while circumventing toxicity to the host cell, which helps to minimize toxic side effects. More importantly, which will become one of the focuses to reduce viral drug resistance.

In future research, we expect to discover more differences in the physical structural properties or surface chemical properties of viral particles and normal cells, which may provide new ‘scopes’ for targeted rupture antiviral nanotherapies. Furthermore, these drugs, which target the virus directly, have the potential to enhance the host's immune response by disrupting viral particles to release viral antigens. In the future, it could be explored whether their use in combination with other antiviral drugs has an immune-enhancing and therapeutic-enhancing effect.

### 6.3 Biomimetic nanotherapy

**Fig. 10:**
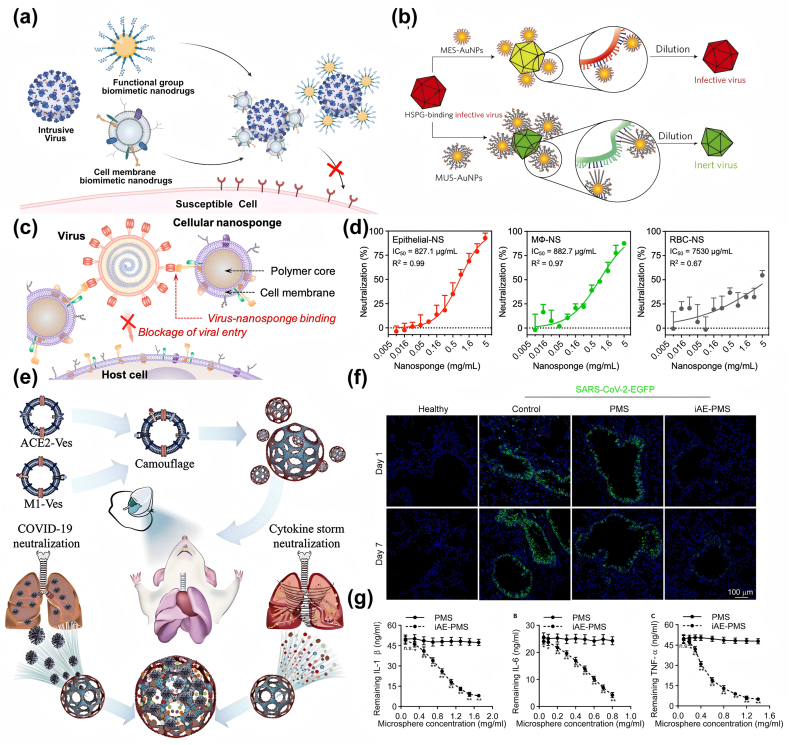
a) Schematic diagram of antiviral nanodrugs in Biomimetic nanotherapy. b) Sulfonate-functionalized inorganic nanoparticles that mimic HSPG inhibit viral binding to the cellular surface HSPG receptor while acting as a virus destroyer. Reproduced with permission [187]. Copyright 2017, Springer Nature. c) Blockage of viral entry and infection through virus-nanosponge binding. d) Cellular nanosponges Epithelial-NS (made from lung epithelial type II cells) and MΦ-NS (made from macrophage membranes) highly neutralize SARS-CoV-2 infectivity, compared with nanosponges made from red blood cell membranes (RBC-NS, used as a control). Reproduced with permission [192]. Copyright 2020, American Chemical Society. Schematic representation of the preparation and mechanism of action of nanodecoys. e) Schematic illustration of the inhaled ACE2-engineered microfluidic microsphere for neutralization of COVID-19 and cytokines. f) Immunofluorescence staining of paraffin sections of mouse lung for pseudotyped SARS-CoV-2-EGFP (green) and DAPI (blue) in healthy, control, PMS, and iAE-PMS groups. Scale bar, 100 μm. g) Inhaled ACE2-engineered porous microsphere inhibited cytokine storm factors. Reproduced with permission [90]. Copyright 2021, Elsevier.

Research on the integration of nanomedicine and bionics is extensive, such as virus-mimetic vaccine vectors [174,175], and biomimetic virus-like particles (VLPs) for immunodiagnostics [176]. In this section, we will emphasize on cellular biomimetic rather than viral biomimetic nanodrugs will analyze their effects as antiviral nanotherapies. The initial phase of viral infection in a host cell is characterized by the binding of a viral particle to a specific receptor located on the cell membrane [177]. So-called cell-based biomimetic nanotherapies are inspired by this infection mechanism. The researchers propose a novel strategy for preventing viral infections and developing antiviral therapies: creating nanomaterials that mimic susceptible cells and bind strongly to viruses, thereby preventing viruses from entering the cells. This section will examine a biomimetic antiviral nanotherapeutic through two lenses (Fig. 10a): first, antiviral nanodrugs that employ functional groups to mimic viral binding receptors, and second, cell-membrane-based antiviral nanodrugs.

#### 6.3.1 Nano-antiviral strategies that mimic virus-binding receptors

As components of the outer layer of the cell membrane and extracellular matrix, sialic acid (SA) and heparan sulfate (HS) serve as common receptors for viral attachment [178]. Both structures are negatively charged and highly expressed in the respiratory and digestive tracts [179]. Viruses such as HSV, HPV and SARS-CoV-2 infect cells via the heparan sulfate receptor [180]. There are also many viruses such as Japanese encephalitis virus (JEV) that promote viral infection by binding to cell membranes via salivary acid receptors [181]. SARS-CoV-2 may also employ SA as a co-receptor or attachment factor for cell entry [182]. Therefore, nanomaterials mimicking SA and HS have been engineered to interfere with viral binding to host cells, resulting in an antiviral effect. Landers et al. [183] evaluated the inhibitory effect of polyamidoamine (PAMAM) dendrimers coupled to multivalent SA on influenza A, completely preventing infection with the H3N2 subtype in a mouse model of influenza pneumonia. Ahn et al. [184] functionalized poly (L-lactic acid)-b-poly (ethylene glycol) (PLLA-b-PEG) diblock copolymer using a sialic acid derivative (methyl-b-neuraminic acid, mNA) and loaded the micelles internally with the antiviral drug amantadine. The mNA-modified micelles function as decoy agents, binding to the haemagglutinin (HA) of the influenza virus, thereby capturing the viral particles and releasing the drug. Papp et al. [185] synthesized SA-functionalized gold NPs (SA-AuNPs), which exhibited high binding activity to host cells through multivalent interactions, thereby inhibiting influenza viruses from binding to them. In addition, the non-toxicity and stability of the SA-AuNPs to cells was also demonstrated.

Sulfonates possess a negative charge and structural similarity to HS, and Sametband et al. [186] thus mimicked HS on the cell surface by means of sulfonated nanomaterials to competitively bound viruses as HS analogues. They introduced a new sulfonate-containing moiety (sulfanilic acid) into GO and found that this partially reduced sulfonated GO (rGO-SO_3_) inhibited HSV-1 infection. Heparan sulfate proteoglycan (HSPG) is a highly conserved target of viral attachment ligands (VAL), so Cagno et al. tried to block viral-cellular interactions by mimicking HSPG in order to develop broad-spectrum antivirals (Fig. 10b) [187]. The synthesis of long linker nanoparticles that mimic HSPG was achieved, resulting in flexible and strong multivalent binding to viruses. The antiviral effects were realized through the generation of forces (∼190 pN) that irreversibly deform viruses. In vitro experiments demonstrated the efficacy against various viruses, including HSV, HPV, respiratory syncytial virus (RSV), and a range of other viruses.

#### 6.3.2 Biomimetic antiviral nanostrategy based on cell membrane

In recent years, the biological properties of cell membrane extracts have been employed for the modification of nanoparticles by encapsulation. It has been discovered that this modification method can increase the circulation time, improve nanoparticle immunocompatibility and enhance the ability to break through the barrier to a certain extent[[188], [189], [190]]. More importantly, host cell membranes have many receptors that recognize viruses, giving specific membrane-derived nanomedicines a strong affinity for them, which has also piqued the interest of researchers in developing membrane-based biomimetic antiviral nanodrugs (Table 1).

**Table 1:** Biomimetic antiviral nanodrugs based on cell membrane.

Nanodrugs	Cell membrane sources	Virus Type	Mechanism of Action	Model Validation	Reference
LSCs nanodecoys	human lung spheroidal cells	SARS-CoV-2	Reducing the viral internalization rate and alleviating inflammatory cell infiltration.	*In vivo* (cynomolgus macaques)	[104]
Epithelial-NS	human lung epithelial type II cells (expressing ACE2)	SARS-CoV-2	The surface of the nanoparticles displays the same protein receptors required for SARS-CoV-2 to enter cells, thereby inhibiting viral entry	*In vitro* (Vero cells)	[192]
MΦ-NS	macrophage (expressing CD147)	SARS-CoV-2	Neutralization of virus	*In vitro* (Vero cells)	[192]
SARS-CoV-2 nanodecoys	engineered 293T/ACE2 cell & human bone marrow monocyte THP-1 (containing GM-CSF)	SARS-CoV-2	Exert a dual-neutralizing effect on viral and inflammatory factors.	*In vitro* (human hepatoma Huh-7 cells, Vero-E6 cells, solutions co ntaining IL-6 and GM-CSF); *In vivo* (acute lung inflammation mice)	[89]
iAE PMS	HEK293-ACE2 cells & macrophages	SARS-CoV-2	Virus blockage and inflammatory cytokine neutralization	*In vitro* (lung fibroblasts); *In vivo* (human ACE2 mice)	[90]
hNTCP-MVs	HepG2 cells	HBV	Interfering with HBV virus binding to host cells and can prevent HBV virus infection	*In vitro* (HBV-Ae/Ba stable cells); I*n vivo* (a human-liver-chimeric mouse model of HBV infection)	[194]
ZIKV-nanodecoys	mosquito cells	Zika	Inhibiting viral replication	*In vitro* (Vero and Hela cells); *In vivo* (type I interferon receptor-deficient mice)	[196]

Angiotensin-converting enzyme 2 (ACE2) is a carboxypeptidase that facilitates the infection of respiratory cells by SARS-CoV-2 through the interaction of spiny proteins (S proteins) [191]. In light of the foregoing, the researchers developed a nanotherapeutic that uses ACE2-containing nanodrugs, which were prepared using ACE2-expressing cell membranes as the raw material, to bind viral particles, thereby preventing them from binding to normal cells.

For example, Li et al. [104] selected human lung spheroidal cells (LSCs) derived from lungs and characterized by high ACE2 expression for the purpose of producing membrane nanovesicles. In vitro experiments demonstrated that LSCs nanodecoys reduced the internalization rate of SARS-CoV-2 virus from 73.8 % to 28 %. In vivo experiments demonstrated a long-lasting retention of LSCs nanodecoys in the lungs (inhalation-delivered mouse model) and the ability to attenuate viral infection-induced inflammatory cell infiltration and lung fibrosis (cynomolgus macaques). In addition to LSCs, some researchers have constructed nanosponges using human lung epithelial type II cells expressing ACE2 and macrophage membranes expressing CD147, which is a co-receptor for SARS-CoV-2 entry (Fig. 10c) [192]. Both types of nanosponges showed strong neutralization of SARS-CoV-2 (Fig. 10d). Besides single-source cell membrane nanomedicines, researchers have created nanomedicines by combining cell membranes from different sources to achieve various effects. Rao et al. [89] fused engineered 293T/ACE2 cell membrane nanovesicles and human bone marrow monocyte THP-1 membrane nanovesicles containing granulocyte-macrophage colony-stimulating factor (GM-CSF) receptors and designed nanodecoys containing enriched ACE2 and cytokine receptors to exert a dual-neutralizing effect on viral and inflammatory factors. Wang et al. [90] genetically engineered HEK-293T cell membranes overexpressing ACE2 receptors and combined pro-inflammatory macrophage membranes capable of broad-spectrum adsorption of inflammatory factors as modifying materials for dual camouflaged microspheres, developing a microsphere inhalation aerosol (iAE PMS) (Fig. 10e), which demonstrated the prevention of SARS-CoV-2 infections (Fig. 10f) and neutralization of pro-inflammatory cytokines (Fig. 10g).

Human sodium taurocholate co-transporting polypeptide (hNTCP) is a transmembrane transport protein that has been proven to bind with high affinity to the HBVpreS protein, a functional receptor for HBV [193]. Liu et al. [194] overexpressed hNTCP receptors in HepG2 cells and subsequently prepared cell membrane vesicles, hNTCP-MVs. In vitro and in vivo antiviral studies have shown that hNTCP-MVs interfere with HBV virus binding to host cells and can prevent HBV virus infection. Furthermore, the researchers encapsulated nanoparticles (TNPs) with CD4^+^ T cell membranes to specifically target HIV viruses that infect T cells. This study confirmed the capacity of the nanoparticles to neutralize HIV-1 strains in multiple instances [195]. Even more amazingly, they found that TNP also selectively binds to cells already infected with HIV and induces autophagy in macrophages, reducing the release of HIV-1 viral particles.

In addition to employing human cell membranes, the researchers have explored the modification of nanoparticles with cell membranes from other susceptible species for certain multi-species susceptible viruses. Rao et al. [196] targeted ZIKV, an infection transmitted by mosquitoes, by coating gelatin nanoparticles with mosquito cell membrane extracts to obtain nanodecoys. This nanodrug has been shown to bind multivalently to the ZIKV virus and inhibit viral replication in cells. In mouse models, their study has shown that nanodecoys coated with extracts from mosquito cell membranes can significantly reduce inflammation caused by viral infections and alleviate fetal microcephaly induced by ZIKV in pregnant mice.

Biomimetic strategies are widely used in nanomedicine, especially in drug delivery applications[[197], [198], [199]], where nanodrugs are surface-modified using functional groups or peptides with high structural similarity to receptor-ligand binding sites to enhance active targeting. Alternatively, nanodrugs are encapsulated with cellular membranes, camouflaged as endogenous cells, to reduce immunogenicity and overcome physiological barriers [200,201]. These biomimetic strategies could also provide a significant advantage in the field of nano-antivirals. Except for the advantages of improved biocompatibility, long circulation, and neutralization of inflammatory factors, we believe that the unique appeal of this membrane biomimetic antiviral nanotherapeutic lies in its capacity to mimic any known host cells targeted by various viruses. Researchers can use molecular biology and genetic engineering to modify multiple susceptible cells of a single virus, or one cell susceptible to multiple viruses, to up-regulate the expression of proteins associated with viral attachment to the cell membrane and prepare nanodecoys. That is to say, this strategy can not only avoid to some extent the efficacy reduction after viral mutation in the case of a single target, but also provide ideas and methods for subsequent research on broad-spectrum antiviral strategies. In addition, with the development of precision medicine and personalized medicine, the preparation of bionic antiviral nanodecoys from isolated patients' own cells is also worthy of research and attention in the future.

### 6.4 PROTAC nanotherapy

**Fig. 11:**
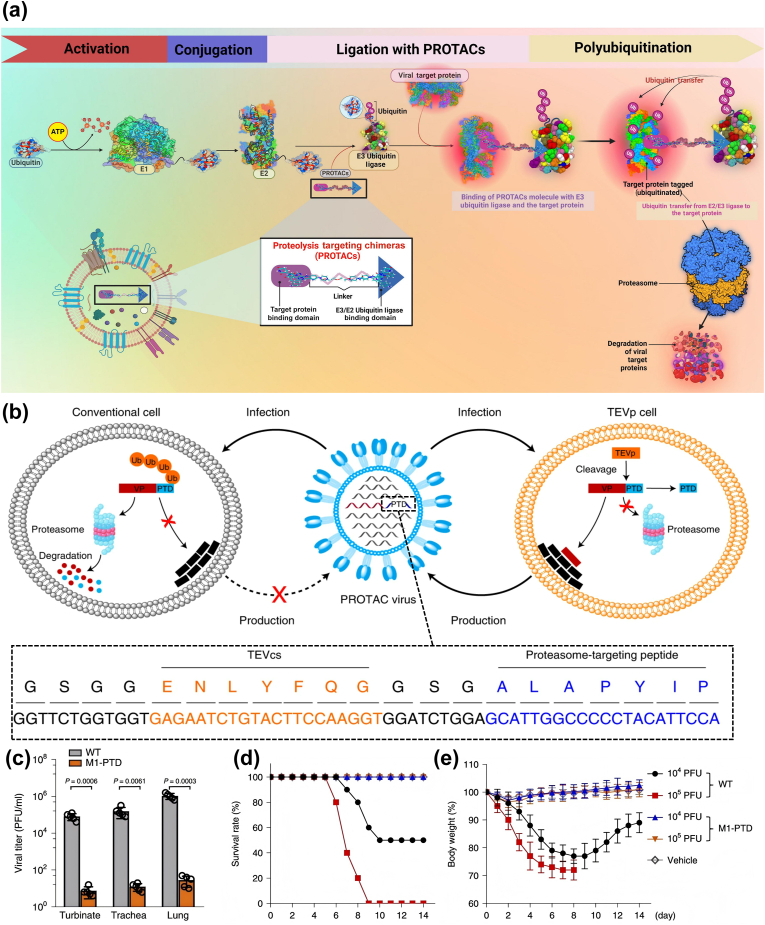
a) Mechanism of action of antiviral PROTACs in degrading viral proteins. Reproduced with permission [225]. Copyright 2024, Elsevier. b) Schematic illustration of the generation of PROTAC viruses and the sequences of amino acids and genes of PTD. c) Viral titers in mouse tissues at day 3 after infection with 10^5^ PFU of WT WSN or PROTAC viruses. d) and e) Survival rates and body weights of mice after intranasal infection with the indicated viruses. Reproduced with permission [224]. Copyright 2022, Springer Nature.

PROTAC is an inventive technology that showcases enormous potential in targeted protein degradation. Its therapeutic efficacy is associated with ubiquitination and proteasome-mediated degradation of targeted proteins. PROTAC molecules are mainly composed of one linker and two ligands, a target protein ligand and an ubiquitin ligase ligand [202,203]. Their distinctive structure enables dual binding to both specific target proteins and E3 ubiquitin ligases, culminating in the formation of a ternary complex. PROTACs enhance the recruitment of E3 ubiquitin ligases to the target proteins, thereby facilitating the transfer of ubiquitin molecules. This process initiates the signaling cascade for proteasomal degradation of the ubiquitin-tagged proteins, thereby revealing the therapeutic potential of PROTACs in the targeted degradation of critical disease-associated proteins [204,205]. These molecules introduce innovative methods for the treatment of diseases ranging from cancer to neurodegenerative disorders, and also encompass utility in viral infections[[206], [207], [208]]. The application of PROTACs to specifically degrade essential viral proteins provides a distinct therapeutic strategy by directly disrupting and hindering the virus's replication process (Fig. 11a).

The advantages of the PROTAC class of drugs are manifold, including the potent and deep degradation of target proteins, the ability to overcome mutation-induced resistance, high selectivity for target proteins shown by the stringent conformational requirements of the ternary complexes, and the reversal of the undruggability of traditional therapeutic target proteins [209,210]. In 2014, Montrose et al. [211] reported on the applicability of PROTAC technology for antivirals, which was one of the first investigations to use PROTAC for virus-related proteins. The PROTAC they developed can induce the degradation of X protein encoded by HBV in liver cells, which is a crucial target protein for HBV replication and participates in the development of HBV-induced liver disease. From then on, the development of PROTAC drugs targeting other viruses and related target proteins also entered a high-speed development stage. For example, the HCV virus NS3 protein has protease and helicase activities, which is crucial for virus replication; only NS3/4A proteases have been considered drug targets, however [212]. Thus, designing a PROTAC that targets NS3/4A for degradation could be a way to inhibit viral replication[[213], [214], [215]]. The VP24 protein of Ebola virus can affect host antiviral defense by inhibiting interferon signaling. Targeting this protein with PROTAC has the potential to restore the interferon-mediated antiviral response and effectively treat viral infections [216]. Xu et al. [217] present novel PROTAC molecules, derived from oseltamivir scaffolds, which demonstrate effective influenza neuraminidase (NA) degradation activity. Notably, they found that this PROTAC exhibited potent antiviral activity against oseltamivir-resistant strains (H1N1, H274Y), which provides a new direction for reversing viral drug resistance.

Nevertheless, it is important to note that the number of antiviral PROTACs currently reported is significantly lower than that for other diseases, such as cancer and neurodegenerative diseases. This discrepancy may be attributed to the constraints of available ligands for targeting viruses. Secondly, as is the case with many small molecule drugs, PROTACs exhibit suboptimal water solubility and tissue permeability due to their molecular composition and structure. The aforementioned factors result in limitations with regard to the absorption, distribution, metabolism and excretion of PROTAC molecules, thus constraining their utilization within the domain of antiviral clinics[[218], [219], [220]]. Consequently, there is a necessity for the integration of nanotechnology and PROTAC. Mukerjee et al. [221] proposed a novel concept involving the use of exosomes as carriers for the delivery of PROTACs against the H3N2 influenza virus in their commentary. They envisioned exosomes reaching the target cell and fusing with the cell membrane, releasing cargo, including PROTAC, into the infected cytoplasm, where PROTAC binds to its target viral protein and recruits the E3 ubiquitin ligase. The subsequent process of ubiquitination of the viral protein leads to its degradation via the proteasome. This ultimately results in the disruption of the reproductive cycle of the influenza H3N2 virus. They also advanced the conjecture of using PROTAC against HIV. By selectively targeting and inducing the ubiquitination and degradation of the Tat protein, which regulates HIV gene expression, this method may effectively disrupt HIV replication and achieve antiviral effects [222]. Due to the circadian rhythmic nature of HIV replication [223], they advanced a proposal to biotechnologically modify exosomes to respond to organismal circadian cues or physiological signals. These signals can be used to indicate the optimal timing for the release of PROTAC drugs synchronized with the host's circadian rhythm in order to administer antiviral therapy in a manner that targets the most vulnerable phases of viral replication.

Furthermore, Si et al. [224] reported an application of PROTAC technology to the production of live attenuated vaccines, whereby live attenuated influenza A virus vaccines are produced by degradation of viral proteins via the endogenous ubiquitin-proteasome system of the host cell. Researchers fused conditionally removable proteasome-targeting domains (PTDs) to a variety of proteins associated with the influenza virus cycle. The PTD contains the proteasome-targeting peptide sequence ALAPYIP and the tobacco etch virus cleavage site (TEVcs). The substrate recognition component of E3 ubiquitin ligase, VHL, recognizes ALAPYIP, leading to polyubiquitination tagging of viral fusion proteins and subsequent proteasomal degradation. The TEVcs linker can be specifically cleaved by tobacco etch virus protease (TEVp), separating the viral proteins from PTDs and avoiding degradation of viral proteins. Researchers initially established a stable cell line expressing the TEVp and subsequently amplified a PROTAC virus harboring the fusion PTD gene in the aforementioned cells. The expression of TEVp results in the cleavage of PTD, thus preventing the ubiquitination and subsequent degradation of viral proteins (Fig. 11b). Consequently, the virus is able to replicate within this cell line, thereby preserving the reproductive potential of the PROTAC virus in the vaccine production process. In contrast, within normal cells, the absence of TEVp results in the degradation of a variety of fusion influenza virus cycle-associated proteins, thereby achieving attenuation. The capacity of PROTAC viruses to elicit potent and extensive humoral, mucosal and cellular immune responses to both homologous and heterologous viruses has been observed in both mouse and ferret models (Fig. 11c,d,e). The employment of the host cell's intrinsic protease mechanism to degrade viral proteins, thereby achieving a state of attenuation, constitutes a novel approach. This method offers a novel concept for the production of live attenuated vaccines for a range of other viruses.

It is noteworthy that there is an absence of experimental literature on the delivery of PROTACs targeting the degradation of virus-associated proteins by means of nanocarriers. However, with reference to the study of nanodelivered PROTACs in oncology therapy, it can be posited that they hold promise in the field of antiviral therapy. Nanocarriers with surface functional modifications and modifications have the potential to deliver antiviral PROTAC to specific sites (e.g. infected cells or potential viral reservoirs), degrading specific virus-associated proteins with high precision while minimizing off-target effects. In addition to the previously mentioned exosome types, the use of other nanocarrier delivery methods such as liposomes with surface modification, microrobots, or microbe-mediated systems warrants experimental investigation as well. In addition, it is imperative to conduct in vivo and in vitro load and release kinetic investigations of various PROTAC drugs, which will provide research ideas and directions for future antiviral nano-PROTAC drugs.

Meanwhile, the study of targeted degradation drugs or PROTAC-derived drugs similar to PROTAC drugs is also noteworthy. For example, pepTACs utilize peptide ligands, which can be engineered to specifically bind to target viral proteins. Similarly, bioPROTACs employ endogenous cellular proteins as recruiting moieties based on the interaction between viral proteins and their cellular cofactors or binding chaperones, inducing degradation of viral proteins. [222] In addition, the unique parasitic properties of viruses make it theoretically possible to target host PROTAC. However, unlike proteins from exogenous viruses, host proteins have essential physiological functions, which means rapid degradation of these proteins may pose some unknown risks. Therefore, the feasibility of antiviral PROTAC targeting host protein degradation remains controversial and more experimental data are needed to validate it.

### 6.5 Gene silencing nanotherapy

**Fig. 12:**
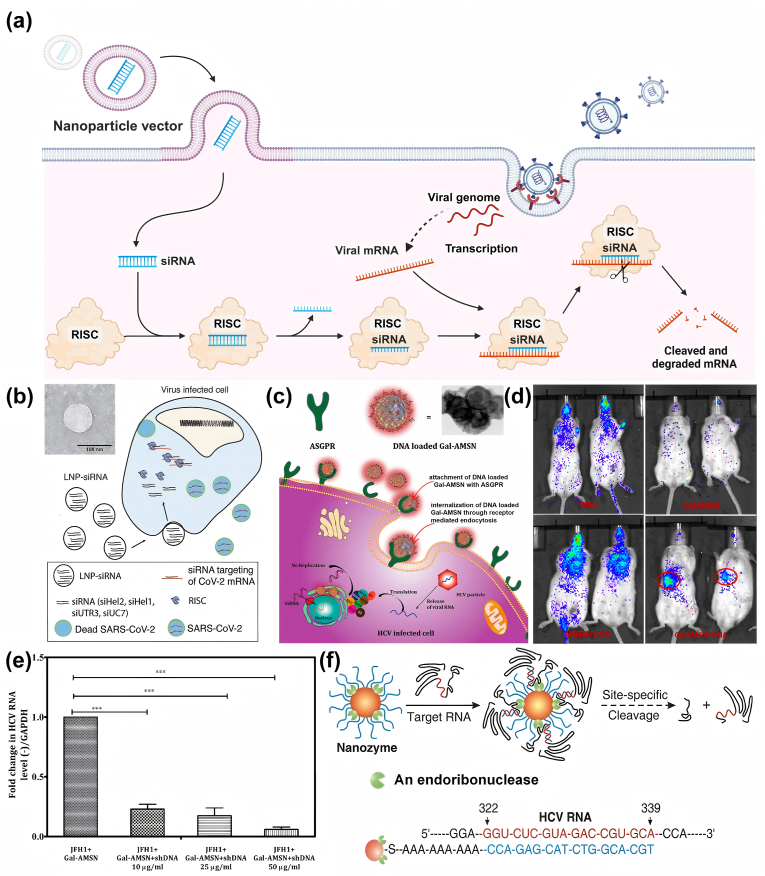
a) Schematic diagram of nanocarrier delivery of antiviral siRNA. Created in https://BioRender.com. b) Delivery of candidate siRNA against SARS-CoV-2 via LNPs. Reproduced with permission [237]. Copyright 2021, Elsevier. c) Mechanism of the action of shDNA-Gal-AMSN complex into HCV-infected cells. d) Biodistribution studies by whole body imaging. e) Antiviral effect of shDNA-Gal-AMSN complex on HCV RNA levels. Reproduced with permission [250]. Copyright 2020, American Chemical Society. f) Schematic representation describing the design and function of the nanozyme with DNA oligonucleotides complementary to the sequence at the HCV RNA position. Reproduced with permission [252]. Copyright 2012, National Academy of Sciences of the United States of America.

RNA interference (RNAi) is a naturally occurring gene-silencing phenomenon in living organisms that involves a series of short fragments of RNA molecules which degrade or inhibit the expression of specific genes [226,227]. Since Mello et al. discovered potent and specific RNA genetic interference in *Caenorhabditis elegans*, researchers have extensively studied RNAi's role in regulating disease-related genes and antiviral defenses, with the goal of applying RNAi in clinical therapeutics [228]. The RNAi process is typically triggered by exogenous double-stranded RNA. This RNA is cleaved by the enzyme Dicer into small segments of small interfering RNA (siRNA) that are 21–23 nucleotides long. The siRNA can bind to the RNA-induced silencing complex (RISC), which has deconjugating and nuclease activities, where one ribonucleic acid strand is removed after being deconjugated and the other guide strand is retained within the RISC and used to target specific mRNAs. The guide strand binds to the target mRNA and directs RISC to either degrade the mRNA or inhibit its translation. This process ultimately results in the downregulation and silencing of the target gene expression (Fig. 12a) [[229], [230], [231]].

In the field of antiviral therapy, viruses can be controlled by silencing viral mRNA at certain stages of infection. However, these nucleic acid-based therapies face significant challenges, particularly in delivering dsRNAs as triggers. Their effectiveness is limited by poor stability in body fluids, rapid degradation from nucleases in the bloodstream, and a negative surface charge that hampers cell entry [232,233]. Fortunately, as nanomedicine develops, the challenges outlined above can be overcome with the help of nanotechnology. In this section, we describe the advanced gene silencing nanodrugs, highlighting the delivery methods of siRNA involving lipid nanoparticle, organic polymers, and inorganic nanoparticles.

#### 6.5.1 Delivery of antiviral siRNA via lipid nanoparticles

Lipid nanoparticles (LNPs) are among the most commonly used drug delivery systems today. [234,235] Due to their enhanced payload, high safety profile, ease of functional group modification, and ability to protect nucleic acid drugs, LNPs are increasingly utilized for siRNA delivery. [236] Idris et al. [237] screened a variety of siRNAs against highly conserved regions of the SARS-CoV-2 virus and attempted to modify the siRNAs with 2′-O-methyl and phosphorothioate to increase stability (Fig. 12b). They demonstrated the potent inhibitory effect of siRNA on pulmonary viruses by encapsulating it in LNP for in vivo injection into mice. Geisler et al. [238] investigated the potential of siRNA-LNP to treat adenovirus infections. For hepatic hAd5 infection, the researchers designed siRNA sipTP, which targets adenoviral precursor terminal protein and features 2′-methyl modifications and phosphorothioate linkages. This siRNA was encapsulated in LNPs containing the cationic lipid XL-10. The results showed that sipTP-LNP treatment significantly reduced hAd5 titers in the livers of Syrian hamsters. Additionally, it decreased liver injury and inflammation, and exhibited anti-hAd5 capacity in groups at different infectious doses. Pei et al. [239] used flexible nano-liposomes to deliver siRNA, named r/si-UL8, which targets the UL8 gene of HSV-1. This approach significantly reduced the viral load in the skin of a preventive mouse model of zosteriform. In addition, effective inhibition of HSV-1 by r/si-UL8 was also reported in a 3D human epidermal skin model. Notably, Pei et al. developed a new tRNA^ser^ scaffold by replacing the tRNA sequence with a 19-nt si-UL8 sequence and its complementary sequences. This innovation facilitates the production of recombinant siRNAs through microbial fermentation and introduces new ideas and methodologies for future siRNA synthesis and preparation.

#### 6.5.2 Organic polymer-assisted siRNA nanotherapy

Due to the negative charge of host cell membranes, some researchers have focused on using cationic organic polymers for DNA/RNA delivery to enhance cellular internalization of nanoparticles through ionic interactions [240,241]. Jamali and colleagues [242] doped siRNA into cationic chitosan to prepare nanoparticles chitosan/siRNA. The results showed that chitosan/siRNA nanoparticles were readily taken up by Vero cells and inhibited influenza virus replication. In addition, the protective effect of chitosan nanoparticles with siRNA delivered through the nasal cavity against influenza virus was also observed in BALB/c mice. Gu et al. [243] prepared anti-HIV nanoparticles composed of chitosan and siRNA, which were functionalized with antibodies through chemical conjugation. They attached antibodies against the transferrin receptor and bradykinin B2 receptor to chitosan nanoparticles (CS-NPs) and delivered siRNAs targeting the SART3 and hCycT1 genes across the blood-brain barrier to inhibit HIV replication in infected brain astrocytes. The experimental results indicated that dual antibody-modified chitosan nanoparticles (Ab-CS-NPs) significantly enhanced cellular uptake and gene silencing efficiencies compared to both unmodified and single antibody-modified chitosan nanoparticles, demonstrating a novel method to improve the viral gene knockdown effect of chitosan-loaded siRNA nanoparticles.

Steinbach et al. [244] reported an anti-HSV-2 infected nano-siRNA formulation for intravaginal administration. Unlike siRNA nanotherapies that target viral genes, they designed siRNAs against host cells to target downregulate the nectin protein, a receptor protein that is present in host cells and is closely associated with viral binding and subsequent transmission. These nanodrugs were produced by encapsulating siRNA in PLGA copolymers and administered intravaginally in a mouse model. The results showed that when exposed to the same lethal amount of HSV-2 virus, mice treated with PLGA-siRNA survived for more than 28 days, a significant increase in survival time compared to untreated mice, demonstrating that nanoparticles are effective in preventing genital HSV-2 virus infection in mice.

#### 6.5.3 Inorganic nanoparticle-assisted siRNA nanotherapy

In order to enhance the delivery and stability of siRNA, some researchers have complexed siRNA with biocompatible inorganic nanoparticles. The multiple functions of these inorganic nanoparticles include enhanced delivery stability, a larger surface area and greater ease of functionalization, demonstrating their advantage of assisting in the delivery of antiviral siRNAs. Li et al. [245] created the Se@PEI@siRNA nanocomplex to target the VP1 gene of the EV71 virus, known for causing hand-foot-mouth disease. This complex is loaded with selenium nanoparticles (SeNPs) and features a surface modified with PEI. The inhibition efficiency of the VP1 gene by Se@PEI@siRNA was more than 70 % in the EV71-infected SK-N-SH neuronal cell line. This study also showed that Se@PEI@siRNA could effectively prevent apoptosis induced by EV71 virus infection in SK-N-SH cells by inhibiting the activation of the apoptosis-related protein Bax signaling pathway. They also investigated the inhibitory effects of PEI and siRNA-modified silver nanoparticles (AgNPs) on enterovirus infection [88]. Ag@PEI@siRNA demonstrated superior ability to enhance cellular uptake and resistance to EV71 virus infection. Similar to Se@PEI@siRNA, Ag@PEI@siRNA also show inhibition on cell death by suppressing caspase-3 mediated cell apoptosis through ROS production. It has been reported that gold nanoparticles can protect siRNAs from degradation by intracellular or extracellular RNase enzymes [246], and thus complexation of AuNPs with antiviral siRNAs has also become one of the research ideas to increase the stability of siRNA delivery. Paul et al. [247] prepared AuNP-siRNA complexes that inhibit DENV infection using a layer-by-layer (LbL) method. AuNP is first coated with an inner layer of HS-PEI (thiol-labelled PEI) via gold-sulfur bonding to make the particles positively charged. Then, negatively charged siRNA was coated on AuNP-PEI through electrostatic interactions, making the complex negatively charged. Afterward, the concentration of PEI in the outer layer was adjusted to change the overall charge from negative to positive. The results showed that positively charged AuNP-siRNA complexes were internalized by Vero cells and significantly reduced DENV2 replication and viral particle release before and after infection. Meanwhile, the RNase-treated AuNP-siRNA complex was still able to inhibit DENV-2 replication in the in vitro model, suggesting that AuNP can protect siRNA from enzymatic degradation and maintain the stability of anti-DENV siRNA. Compared with other liver-targeted nucleic acid delivery vehicles such as liposomes and cationic polymers, mesoporous silica nanoparticles (MSNs) have the advantages of higher surface area, controllable pore size, and ease of functionalization, making them a promising nucleic acid nanocarrier that has been widely utilized [248,249]. Mukherjee et al. [250] delivered shDNA via galactose- and amine-coupled mesoporous silica nanoparticles (Gal-AMSN) to treat hepatitis C infection (Fig. 12c). Therapeutic shDNA acts as a precursor for the in situ generation of siRNA that targets the HCV 5′-untranslated region (5′-UTR) to achieve antiviral effects. The results indicate Gal-AMSN-shDNA is specifically localized in the liver (Fig. 12d), and its use in HCV-JFH1 cell culture resulted in a significant 94 % reduction in viral RNA levels (Fig. 12e).

#### 6.5.4 Other RNAi nanotherapy

Sun et al. [251] designed siRNAs targeting highly conserved regions of SARS-CoV-2 and constructed siRNA nanoparticles using RNA self-assembly technology. In contrast to previously mentioned carrier-based siRNA delivery, this self-assembling nucleic acid nanotechnology enhances delivery efficiency and may reduce the risk of cytotoxicity and adverse immune responses caused by the use of nanocarriers. Cells efficiently internalize self-assembled RNA nanoparticles via the endocytosis pathway. The result showed that after 24 h of incubation, the nanoparticles could be detected in 95 % of the cells and significantly inhibited SARS-CoV-2 virus replication.

In addition to triggering RNAi through the introduction of exogenous double-stranded RNA (dsRNA or siRNA), gene silencing-associated antiviral therapies can also be performed by designing drugs to directly induce cleavage of target RNAs. Cao et al. [252] reported an artificial nanoparticle complex that mimics the function of the cellular RISC mechanism to induce target RNA cleavage (Fig. 12f). They designed an HCV nanoenzyme that actively cleaves HCV RNA fragments containing the 5′-UTR in a sequence-specific manner to achieve antiviral effects. The 5′-UTR region is highly conserved and contains an important structure that controls the initiation of HCV RNA translation, known as the internal ribosome entry site. The results showed that the nanoenzymes were resistant to proteases, reduced HCV RNA levels by over 99 % in treated mice, and exhibited enhanced antiviral activity against HCV in both in vivo and in vitro models.

RNA interference has emerged as an effective strategy for precisely eliminating disease-causing agents, particularly in viral infections. Exploring additional siRNAs that target the silencing of the virus's own genes can help mitigate the impact on normal cells and genes. The design and synthesis of siRNAs are straightforward. Even small viral genomes can offer multiple targeting regions, making this strategy applicable to a wide range of antiviral applications. In addition, by investigating siRNAs that target conserved viral sequences, it is possible to target multiple strains of the virus (including mutant strains with unchanged conserved sequences), achieving broad-spectrum activity while providing a solution to potential drug resistance. As an emerging antiviral strategy, attention should be paid to optimizing the delivery of nucleic acid-based drugs in the future clinical translation process to maximize the maintenance of drug stability and avoid interference from many in vivo substances, such as degradation by nucleases. At the same time, optimizing the siRNA design methods to avoid miRNA-like off-target effects of siRNAs that lead to the degradation of multiple unwanted mRNAs is essential for the successful treatment of diseases [253]. Additionally, exploring the co-delivery of multiple siRNAs is crucial. This could involve either the combination of siRNAs targeting various viral gene fragments of a single virus or the co-delivery of different siRNAs targeting both viral genes and viral cycle-related host cell genes to explore the enhancement of viral suppression in a coordinated manner.

### 6.6 Assisting nanotherapy: nanodrugs for complementing in the treatment of viral infections

In treating viral infections, researchers are exploring not only antiviral strategies that directly target and destroy the virus but also complementary and assisting approaches that could enhance treatment efficacy. There is an urgent need for this indirect and supplementary treatment for viral infections, particularly for patients already infected with the virus. Adjusting the immune response can help to further control viral infections, while addressing the inflammation they cause can prevent critical symptoms. This section outlines assisting nanotherapies, focusing on assisting-immunological nanodrugs and those designed to manage inflammation after viral infections.

#### 6.6.1 Assisting-immunological nanodrugs

Interferons (IFNs) are a class of important cytokines secreted by host cells in response to viral infections or immune stimulation. They are classified into three types: type I (IFN-α/β), type II (IFN-γ), and type III (IFN-λ) [254]. Interferon has diverse antiviral effects. It induces the expression of proteins that block key aspects of viral replication, enhances the immune activity of natural killer cells and T cells, and coordinates innate and adaptive immune responses [255,256]. This helps limit viral spread and facilitates the clearing of infection. Thus, even though interferon does not directly kill the virus, it plays a key role in coordinating antiviral immunity and assists antiviral therapy.

**Fig. 13:**
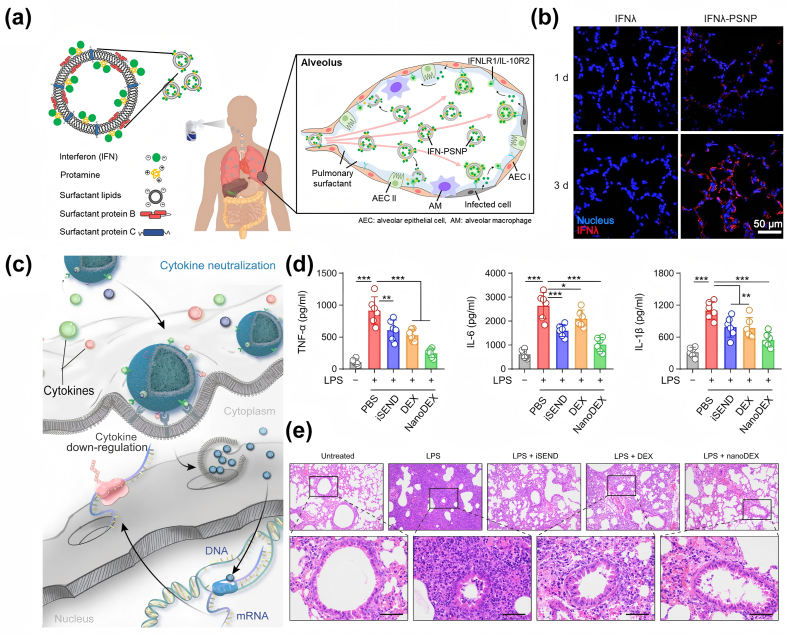
a) Schematic diagram of inhalable IFNλ-PSNP and b) confocal fluorescence images of alveolar region of the lungs 1 day and 3 days after IFNλ-PSNP inhalation: nucleus (blue, Hoechst), IFNλ (red). Reproduced with permission [258]. Copyright 2024, American Chemical Society. c) Schematic illustration of inhalable nanoDEX and the two-step strategy against COVID-19 cytokine storm: cytokine down-regulation by DEX and cytokine neutralization by the iSEND. d,e) nanoDEX reduced cytokine levels, significantly suppressed the LPS-induced severe lung injury characterized by alveolar cavity disappearance, alveolar wall incrassation, inflammatory cell infiltration, and vascular dilatation and congestionin an acute pneumonia mouse model. Reproduced with permission [269]. Copyright 2023, American Association for the Advancement of Science.

IFN-λ activates local antiviral responses by interacting with mucosal-specific receptors [257]. Its unique role in barrier tissues, including the respiratory and intestinal tracts, makes it an important target for developing new mucosal antiviral therapies. Kim et al. [258] created inhalable nanoparticles loaded with IFN-λ and lung surfactant, called IFNλ-PSNP, for treating lung infections caused by the influenza A virus (Fig. 13a). They found that inhaled IFNλ-PSNPs effectively localized IFN-λ to the alveolar region and significantly limited IAV replication in the lungs compared to mice administered recombinant IFN-λ only (Fig. 13b). IFNλ-PSNPs not only more effectively reduced the viral RNA levels but also result in the increase of monocyte frequency in concert with restoration of T and B cells composition. More importantly, mRNA levels of related interferon-stimulated genes (ISGs) were also significantly altered, with the transcriptional profiles of monocytes shifted toward heightened IFN responses following IFNλ-PSNP treatment. This proposed inhaled interferon nano-formulation significantly enhances IFN-λ delivery to the lungs. It also provides a new strategy for triggering an effective and assistant antiviral immune response during respiratory viral infections. Notably, the research by Kim et al. revealed that interferon nanocarriers are more advantageous than using directly administered interferon. These advantages consist of more efficient delivery and targeting, faster and stronger antiviral immune responses, and more significant protective effects, which also provide a feasible guidance and direction for research on interferon-assisted antiviral therapy. In the future, we can explore various avenues for IFN-loaded nanoparticles, including surface modification for better targeting, the delivery of multiple drugs or immunomodulators to achieve synergistic antiviral effects and enrich assisting antiviral therapies.

Besides research on exogenous IFN nanocarriers, investigations into nanoagonists that trigger the production of endogenous IFN have led to significant insights for assisting antiviral therapies. It has been reported that Mn^2+^ activates type I interferon (type-I-IFN) expression and is an IFN stimulator, but its application is limited by non-specific distribution and neurotoxicity [259]. Therefore, our team developed a manganese nanodepot (nanoMn) for enhancing host antiviral immune responses [93]. NanoMn has a core made of manganese phosphate and a shell composed of polyethylene glycolated phospholipids. This structure enhances cellular uptake and allows for the pH-sensitive release of Mn^2+^. In vitro experiments revealed that nanoMn effectively inhibits a broad spectrum of viruses, including VSV-GFP, Mouse Hepatitis Virus-A59, HSVs and LCMVs. Compared to free Mn^2+^ (EC_50_ = 71.67 μM), nanoMn showed a higher potency in the inhibition of viral replication (EC_50_ = 3.897 μM). In a mouse model, we found that nanoMn targets and polarizes macrophages, leading to the production of large amounts of type I interferon. It also recruits large numbers of monocytes to local inflammatory foci, enhances antiviral immune responses, and attenuates coronavirus-induced tissue damage. In addition, we found that nanoMn promotes antigen delivery and enhances virus-specific T cell differentiation and adaptive immune responses. Its potential as an immune adjuvant is encouraging, especially considering its high safety profile and lower risk of inducing neurotoxicity from manganese ions. In conclusion, nanoMn offers a simple, safe, and potent strategy with potential for antiviral therapy and prophylaxis. In the field of tumor immunity, there is a wealth of significant research on stimulating interferon production through mechanisms such as TLR9 [260], STING [261], and RIG-I [262], which provides ideas and implications for assisting antiviral therapies. These two therapeutic scenarios share commonalities, as both require the activation of immune cells such as T-cells and NK cells and induce the expression of ISGs. Therefore, it is worthwhile to try to develop antiviral nanodrugs in the future by drawing on the findings of related tumor immunity.

In addition to nanostimulants and IFN nanocarriers, the research on multifunctional nanodrugs that can stimulate the production of interferon is also promising. For example, Liu et al. [263] developed a nanodrug called CoVR-MV. This drug not only interferes with the binding of viruses to host cells via surface receptor proteins, but also effectively facilitates the internalization of viruses by macrophages and promotes their removal through phagocytosis. More importantly, CoVR-MV also deregulated inhibition of interferon regulatory factor 3 (IRF3) activation and promotes a swift production and signaling of endogenous type I interferon. It can be said that CoVR-MV has multiple effects: it directly neutralizes viruses, enhances macrophage phagocytosis, and stimulates endogenous interferon production, thus fulfilling both direct antiviral and immune-assisting roles.

From nanoformulations of exogenous IFN to multifunctional nanodrugs that activate the generation of endogenous IFN, these drugs do not yet target the virus directly but have the potential to be developed as assisting-immunological antiviral drugs. Such drugs also provide new ideas for the study of antiviral nanodrugs. We hope that in the future, more assisting antiviral methods with immune activation will be proposed, providing clinicians with more effective options for controlling viral infections through synergistic direct antiviral strategies.

#### 6.6.2 Controlling post-viral infectious inflammatory nanodrugs

Inflammation and oxidative stress caused by viral infections can exacerbate tissue damage and, in more severe cases, cause a cytokine storm that can result in multi-organ failure or even death, which is worthy of high priority in antiviral therapy. Some nanodrugs that reduce the inflammatory response caused by viral infections are also of importance.

During the course of COVID-19 pandemic, a significant number of patients succumbed to acute respiratory distress syndrome (ARDS), a severe manifestation of viral pneumonia [264,265]. Dexamethasone (DEX) is the first drug proven to save lives in patients with severe COVID-19 [266]. However, being a glucocorticoid, it has receptors that are widely expressed in many cell types. Consequently, its use can lead to serious side effects, including osteoporosis, fractures, and ischemic necrosis of the femur [267,268]. Rao et al. [269] have developed a novel delivery vehicle based on cholesterol-engineered neutrophil nanovesicles (iSEND) for the inhalation delivery of DEX, aiming to target inflamed lung tissue, enhance the therapeutic efficacy and reduce side effects in severe COVID-19 cases. Thanks to the inhalation route of administration and the abundance of chemokine receptors on neutrophil membrane nanovesicles (N-NVs), nanoDEX showed enhanced retention in inflamed lungs and responded to the COVID-19 cytokine storm through DEX-induced cytokine downregulation and N-NVs-induced neutralization (Fig. 13c). Researchers tested the anti-COVID-19 efficacy of inhalable nanoDEX using multiple animal models. In comparison to DEX treatment, nanoDEX effectively inhibited the upregulation of cytokines and chemokines induced by SARS-CoV-2 infection (Fig. 13d). Furthermore, it increased the expression of genes associated with inflammation and tissue repair, while decreasing the expression of genes in the apoptotic pathway. This shows great potential for COVID-19-associated immune dysregulation and lung injury (Fig. 13e), and provides new ideas for the treatment of COVID-19.

Peng et al. [270] developed a cerium-based tannic acid (CeTA) nanoenzyme and combined it with a self-assembling peptide to create CeTA-K1tkP, a nasal inhalation product for treating viral pneumonia. In inflamed regions with high ROS levels, the thioketal linker on CeTA-K1tkP is cleaved, leading to the detachment of hydrophilic PEG, the aggregation of the peptide into β-sheets, and the formation of fibrous structures. These structures are targeted to the inflammation site in large quantities and form aggregates. Meanwhile, the CeTA nano-enzymes in these structures have multi-species SOD and CAT enzyme functions that can efficiently scavenge ROS and alleviate inflammation. In a mouse model of H1N1 viral pneumonia, researchers found that CeTA-K1tkP aggregates in inflamed tissues, which enables the nano-enzymes to effectively decompose ROS and facilitate macrophage polarization to the inflammation-suppressing M2 type. In addition, the researchers found that CeTA-K1tkP respectively binds to the HA protein of the H1N1 virus and the haemagglutinin-neuraminidase (HN) protein of the Sendai virus (SeV) and has a certain broad-spectrum virus-neutralizing effect. Therefore, the CeTA-K1tkP nanoplatform offers a novel solution for the treatment of various deep inflammatory diseases caused by viral infections. Currently, nanoenzymes are a prominent topic in anti-inflammatory research. Their potential applications in treating viral inflammation warrant further investigation, and the research conducted by Peng et al. provides an excellent entry point. Nanoenzymes that demonstrate high anti-inflammatory activity in vitro have the potential to become the next generation of antiviral nanomedicines. However, achieving this goal requires improved drug delivery methods, effective ways to neutralize viruses, enhanced immune responses, and other multifunctional improvements.

Viral infections can lead to various complications, with the type and severity depending on the virus type, the infection site, the host's immune status, and how promptly treatment is administered. For antiviral strategy, there is also a need to develop nanodrugs for assisting therapy, such as boosting the body's immunity against antivirals and reducing inflammation caused by viral infections. Integrating emerging nanotechnologies—such as biomembrane-encapsulated delivery systems, environmentally intelligent responses, and self-assembly—with the therapeutic needs of viral infections may lead to new strategies for developing vaccine adjuvants and treating inflammatory diseases.

## 7 Challenges encountered in antiviral nanostrategies

It has to be acknowledged that the outbreak of COVID-19 has led to increased investment and development of antiviral drugs worldwide, undoubtedly providing a stockpile of strategies to combat the viral epidemic. Currently, in addition to focusing on the development of new drugs and vaccines, numerous strategies employing nanodrugs have also been proposed. Despite the significant efforts invested by researchers in the application of antiviral nanoparticles, with numerous strategies and scientific publications proposed, a substantial proportion of nanomedicines that have been subjected to clinical trials have yielded unsatisfactory results, and there is an even paucity of examples of successful commercialization [271,272](Table 2). It has to be admitted that there are still some challenges and limitations in the clinical translation of nanodrugs, and there are still many problems to be solved in the future.

**(i) Clinical efficacy assurance and safety validation:** Most antiviral nanodrugs reported in scientific articles have demonstrated excellent antiviral efficacy in vitro using mammalian cell line models and in rodent models. However, before they can enter clinical trials, further testing is necessary in animal models that closely replicate human disease responses, such as ferrets. Additionally, models with a high degree of genetic similarity and comparable physiological and anatomical characteristics to humans, like rhesus macaques and other primates, should also be utilized. Antiviral nanoparticles may cause harmful or complex reactions in humans. Therefore, further evaluation of nanomedicines for biocompatibility, immunogenicity, safety, and effective antiviral properties in animal models and clinical trials is essential.

**Table 2:** Summary of clinical translation cases of nanoscale antiviral drugs.

Nanodrug	Virus Target	Mechanism of Action	Name	Company/Institution	Applicable population	Clinical Stage	Time	References
Lipid Nanoparticles	SARS-CoV-2	mRNA encoding viral spike protein to induce immunity	mRNA-1273	Moderna	Adults (≥18 years)	Approved	2020–2021	[285,286]
			BNT162b2	Pfizer-BioNTech	Adults (≥18 years; later expanded to children)	Approved	2020–2021	[287]
	HBV	siRNA targeting viral RNA to inhibit replication	ARC-520	Arrowhead Pharmaceuticals	People with high viral load	Terminated	2022–2023	[288]
	RSV	mRNA encoding viral fusion protein for immune activation	AREXVY	GSK	Older Adults	Approved	2023	[289]
			MRESVIA	Moderna			2024	[289]
Nano-crystals	HIV-1	Extended drug half-life via slow-release nano-crystals; inhibits viral integrase	Cabotegravir	ViiV Healthcare	High-risk HIV population	Approved	2021	[290]
Protein Nanoparticles	SARS-CoV-2	Display viral antigens to activate T/B-cell immunity	NVX-CoV2373	Novavax	Adults (≥18 years)	Approved	2021–2022	[291]
Viral Nanoparticles	HBV	Delivering HBV antigens to induce significant HBV-specific T-cell immune responses.	VTP-300	Barinthus Biotherapeutics	Patients with chronic hepatitis B who have viral suppression.	Phase I/II	2023	[292]

Acute and chronic toxicological studies are of paramount importance in the clinical translation of nanomedicines. While the nano-size confers unique potency to the drug, it also increases the likelihood of interactions (e.g., aggregation, decomposition) with normal cells and various constituents in the body due to the changes in conformation, the increase in surface area and surface charge, which may result in toxicity [273]. Therefore, long-term monitoring of the potential toxicity and metabolism of nanodrugs in the body is needed, including studies of how nanoparticles interact with the biological environment to ensure that they do not pose a threat to the human body. This requires advancements and enhancements in analytical techniques for in vivo metabolism, such as real-time monitoring and imaging. In addition, dose-related toxicity must be considered and the appropriate dose range for nanodrugs must be carefully determined. We recognize that nanodrugs with effective antiviral activity may carry some risk; thus, researchers will need to model and evaluate the risk-benefit ratio to determine whether the need for treatment is worth the risk. Toxicology based on nanodrugs or nanocarriers is a relatively new discipline, but it deserves the attention of all researchers and requires in-depth exploration of its aspects to ensure the safe manufacture and use of nanodrugs.

**(ii) The challenge of drug resistance**:For viruses with high mutation rates, viral resistance to drugs due to mutations and target changes that often occur during replication is a key challenge that researchers must address [274,275]. Many teams are focusing their R&D on broad-spectrum antivirals, and the key and the difficulty is to find the intersection and balance between specificity and broad-spectrum. Specificity is defined as targeting drugs at viral particles or infected host cells, which reduces their negative effects on normal tissues. Broad-spectrum strategies require developers to focus on the highly conserved structures of the virus to ensure that the drug's target can withstand variations in the viral genome, thus helping to prevent the emergence of drug resistance. Good targets for intervention include lipid bilayers that envelop viruses, as described in section 6.2. In addition, the enhanced immune-adjuvant properties (e.g., stimulation of IFN production) exhibited by some NPs, as described earlier, provide a strong complement to address viral tolerance. We believe that the prospect of exploring broad-spectrum antiviral targets from a nanoscale perspective to avoid drug resistance is promising and worthwhile.
**(iii) Mass-production and commercialization challenges:** Surface functionalization of nanodrugs can improve their targeting and selectivity to reduce dosage and side effects; however, it is worth noting that functionalization also increases the number of nanodrugs manufacturing steps, resulting in the cost escalating with the technical difficulty. In addition, formulation stability must be ensured during storage and distribution of nanodrugs as commodities, which requires rigorous research to explore the ideal storage conditions for different nanotechnology-based products [97,276]. To address the challenges mentioned above, companies and research institutions need to strengthen future communication, establish a joint cooperation model from R&D to clinical industrialization, and develop strategies for scaling up nanomedicine production that are more affordable, yield higher outputs, and improves environmental sustainability.
**(iv) Establishment of a standardized quality assessment system:** With the rise of nanotechnology, numerous companies around the globe have started producing nanomaterials. Disappointingly, reports indicate that the absence of relevant laws and regulations makes it difficult for the quality of these nanomaterials to meet the standards for most applications [277]. Therefore, it is crucial to introduce appropriate industry standards and norms to establish GMP (Good Manufacturing Practice of Medical Products) for the production of nanodrugs [278]. In the future, we can ensure the consistency and safety of mass-manufactured products by establishing a comprehensive, detailed, and standardized quality evaluation and monitoring system. This will help high-quality products stimulate the market and boost commercialization.

Clinical translation of antiviral nanomedicines is a multifaceted challenge that includes technical, regulatory, and market aspects. Moreover, addressing these challenges requires the combined efforts of researchers, clinicians, government regulatory agencies, and pharmaceutical manufacturers. Through interdisciplinary and multisectoral collaboration, we believe that more innovative and effective antiviral strategies and therapies at the nanoscale can be stimulated, providing a technological reserve addressing potential future global pandemic viral infections.

## 8 Summary and future outlook

Developing novel antiviral nanodrugs is important and necessary to combat viral outbreaks and potential pandemics. We summarized the clear advantages of nanomedicines for antiviral therapy, including more novel mechanisms of action, broad-spectrum antiviral potential, overcoming mutation-induced resistance and enhanced in vivo distribution. Meanwhile, in this review, we elaborated on the current research of advanced antiviral nanodrugs according to the classification of nanotherapy (Table 3), such as light-mediated antiviral nanotherapies, RNA interference-based gene silencing nanotherapies, and biomimetic antiviral nanotherapies that mimic biological membranes or receptors, PROTACs-mediated nanotherapies, etc. We suggest future directions for developing antiviral nanomedicines by critically reviewing various nanotherapies, with the aim of stimulating researchers to investigate new ideas.

**Table 3:** Summary of antiviral nanomedicines under various nanotherapies in this review.

Treatment Strategy	Nanodrug	Virus Type	Mechanism of Action	Model Validation	Reference
Light nanotherapy	PPIX-MWNT	Influenza A/X-31 (H3N2)	Light-induced inactivation by the production of ROS	*In vitro*	[118]
	SMRGO	HSV-1, EV71, and Influenza virus H9N2	Photothermal effect causing viral protein denaturation and nucleic acid damage	*In vitro* (Vero African green monkey kidney epithelial cells)	[149]
	Ag/TiO2	H1N1 and EV 71	ROS generation leading to viral inactivation	*In vitro*	[156]
	CuxO-TiO2	H1N1 Influenza A virus and bacteriophage Qβ	Photocatalytic generation of ROS and oxidation of viral components	*In vitro*	[157]
	ZnPc-UCNPs	DENV2 and Ad5V	NIR light-induced ROS generation for viral inactivation	*In vitro* (HepG2 cells); *In vivo* (mouse model)	[162]
Targeted-rupture nanotherapy	GPS6.8	VSV	Disruption of viral envelope by targeting low pH	*In vitro* (HeLa cells,H460 cells, acidified model lipid membranes); *In vivo* (C57BL/6 mouse model)	[109]
	AH-D	ZIKV	Targeting high curvature of viral envelope to disrupt it	*In vitro* (liposomes); *In vivo* (mouse model)	[167]
Biomimetic nanotherapy	SA-PAMAM	Influenza A subtypes (H1N1, H2N2, and H3N2)	Inhibiting viral adhesion and infection of cells	*In vivo* (a H3N2 subtype in a murine Influenza pneumonitis model)	[183]
	mNA-PLLA-b-PEG	Influenza A virus	Capturing the viral particles and releasing the drug	*In vitro*	[184]
	SA-AuNPs	Influenza A strain X31	Inhibiting viral adhesion and infection of cells	*In vitro* (red blood cells)	[185]
	rGO-SO3	HSV-1	Competitively bind viruses as HS analogues	*In vitro* (Vero cells)	[186]
	HSPG-NPs	HSV, HPV, RSV, Dengue and Lenti virus	Multivalent binding leads to local distortions and eventually to a global virus deformation	*In vitro* (human cervicovaginal histocultures infected by HSV-2); *In vivo* (in mice infected with RSV)	[187]
	LSCs nanodecoys	SARS-CoV-2	Reducing the viral internalization rate and alleviating inflammatory cell infiltration.	*In vivo* (cynomolgus macaques)	[104]
	Epithelial-NS	SARS-CoV-2	The surface of the nanoparticles displays the same protein receptors required for SARS-CoV-2 to enter cells, thereby inhibiting viral entry	*In vitro* (Vero cells)	[192]
	MΦ-NS	SARS-CoV-2	Neutralization of virus	*In vitro* (Vero cells)	[192]
	SARS-CoV-2 nanodecoys	SARS-CoV-2	Exerting a dual-neutralizing effect on viral and inflammatory factors	*In vitro* (human hepatoma Huh-7 cells, Vero-E6 cells, solutions co ntaining IL-6 and GM-CSF); *In vivo* (acute lung inflammation mice)	[89]
	iAE PMS	SARS-CoV-2	Inhibiting viral adhesion and infection of cells	*In vitro* (lung fibroblasts); *In vivo* (human ACE2 mice)	[90]
	hNTCP-MVs	HBV	Interfering with HBV virus binding to host cells and can prevent HBV virus infection	*In vitro* (HBV-Ae/Ba stable cells); I*n vivo* (a human-liver-chimeric mouse model of HBV infection)	[194]
	ZIKV-nanodecoys	Zika	Inhibiting viral replication	*In vitro* (Vero and Hela cells); *In vivo* (type I interferon receptor-deficient mice)	[196]
Gene silencing nanotherapy	sipTP-LNP	hAd5	Delivery of siRNA that targets adenoviral precursor terminal protein, inhibit viral replication	*In vivo* (hAd5-infected immunosuppressed Syrian hamsters)	[238]
	dmLNP-siRNAs	SARS-CoV-2	Delivery of siRNA to interfere with RdRp/Hel/5′UTR synthesis	*In vitro* (Vero and HEK293 cells); *In vivo* (K18-hACE.2 mice)	[237]
	r/si-UL8	HSV-1	Delivery of siRNA that targets the UL8 gene of HSV-1, inhibiting viral replication	*In vitro* (3D human epidermal skin tissue model); *In vivo* (HSV-1 mouse zosteriform model)	[239]
	chitosan/siRNA	Influenza	Delivery of siRNA against influenza nucleoprotein, inhibiting viral replication	*In vitro* (Vero cells); *In vivo* (BALB/c mice)	[242]
	Ab-CS-NPs	HIV	Delivery of siRNAs targeting the SART3 and hCycT1 genes across the blood-brain barrier to inhibit HIV replication in infected brain astrocytes	*In vitro* (hCMEC/D3 and U138-MG cells)	[243]
	PLGA-siRNA	HSV-2	Delivery of siRNAs against host cells to target downregulate the nectin protein	*In vitro* (HeLa cells); *In vivo* (C57BL/6 mice)	[244]
	Se@PEI@siRNA	EV71	Targeting EV71 VP1 gene, restrain the apoptosis signaling induced by Bax	*In vitro* (nerve cell line SK-N-SH)	[245]
	Ag@PEI@siRNA	EV71	Decreasing the apoptotic cell population, preventing the spread of the EV71 virus	*In vitro* (Vero cells)	[88]
	AuNP-siRNA	DENV-2	Resistance to RNase and enhanced stability of targeted viral gene siRNAs	*In vitro* (Vero cells)	[247]
	Gal-AMSN-shDNA	HCV	Targeting the HCV 5′-untranslated region (5′-UTR) to achieve antiviral effects	*In vitro* (HCV-JFH1 cell); *In vivo* (BALB/c mice)	[250]
	RNA self-assembly NPs	SARS-CoV-2	Targeting highly conserved structural and non-structural protein coding regions in the SARS-CoV-2 genome	*In vitro* (Caco-2-N cell)	[251]
	HCV nanoenzyme	HCV	Cleaving HCV RNA fragments containing the 5′-UTR in a sequence-specific manner	*In vitro* (Huh7.5 cells,FL-Neo cell line);*In vivo* (NOD/SCID mice)	[252]
Assisting nanotherapy	IFNλ-PSNP	IAV	Inducing rapid antiviral immune responses in the lung	*In vivo* (C57BL/6J)	[258]
	nanoMn	VSV HSV LCMV	Inducing of interferon production	In vitro (HeLa cells, BMDMs); In vivo (Irf3 −/−mice)	[93]
	CoVR-MV	SARS-CoV MERS-CoV SARS-CoV-2	Absorbing the viruses, facilitating the internalization of viruses by macrophages, stimulating endogenous interferon production	*In vitro* (H1299-hACE2); *In vivo* (Syrian hamster)	[263]
	nanoDEX	SARS-CoV-2	Inflammatory control of viral pneumonia with cytokine downregulation	*In vitro* (RAW 264.7); *In vivo* (acute pneumonia mice, osteoporosis rat,K18-hACE2 mice, rhesus macaques)	[269]
	CeTA-K1tkP	H1N1 SeV	Scavenging ROS modulates inflammatory response and neutralizing viruses	*In vitro;In vivo* (H1N1/SeV-mediated pneumonia mice,virus-bacteria co-infection pneumonia mice)	[270]

While numerous nanoscale-based antiviral strategies have been proposed, their development remains confined to laboratory studies, and the therapeutic application and clinical translation is still in early stages. We perceive the following two major directions for the future development of antiviral nanomedicines.

①Overcoming challenges and limitations in the field of nanomedicine development by the utilization of novel technologies. The rapid development of materials science, artificial intelligence and other disciplines is also expected to provide a new boost for the development of antiviral nanodrugs in the future. Using techniques such as micromachining and tissue engineering, researchers can build organ chips in vitro. This organ chip has the capacity to simulate the physiological conditions of human organs, thereby facilitating the study of the metabolic processes of new drugs, the toxic effects of drugs, and the evaluation of drug efficacy [279,280]. In the future, the utilization of organ chips will serve as a substitute for animal models in clinical trials, thereby significantly enhancing the efficacy of pharmaceuticals and the efficiency of research and development processes. The employment of machine learning algorithms facilitates the simulation of nanomedicine-virus interactions and the prediction of the structure-function relationship of nanomedicines [281]. This can contribute to the acceleration of the R&D process and the reduction of R&D costs. For instance, Marcelo et al. [282] utilized AI to achieve molecular docking simulation against the SARS-CoV-2 spike protein receptor-binding domain (RBD) and synthesized AI bio-inspired peptides. In light of the ongoing advancements in deep learning and big data extraction algorithms, there is a strong possibility that, in the future, artificial intelligence will be capable of predicting viral mutations. This would provide a significant contribution to the efforts to prevent viral pandemics.
②Undertaking active exploration of novel ways and methodologies for clinical translation of nano-antivirals. We acknowledged that, as a new class of pharmaceutical agents, these antiviral nanoparticles taken into the body require rigorous scrutiny during both clinical trials and approval for marketing. However, researchers could adopt a different perspective; it is not a requirement for all NPs to exert antiviral efficacy only in vivo. For instance, the elimination of circulating viral particles from the blood of infected subjects during in vitro blood purification relies on advances in medical device technology, which provides a new arena for nano-antiviral drugs. Kang et al. [283] investigated magnetic nanoparticles functionalized by mannose-binding lectin (MBL), which can bind to sugar molecules on the surface of various pathogens such as bacteria and viruses [284], for blood purification applications. MBL-NPs are mixed with circulating blood in vitro, where they bind to the virus, and then the NPs-pathogen mixture can be magnetically separated before the blood is returned to the host. Nanomedicines may bring new innovations to medical device technology through this in vitro approach, and similarly NPs-enabled medical devices provide a new pathway for clinical translation of antiviral nanodrugs.

Currently, although antiviral nanodrugs cannot shake the dominant position of small molecule drugs at present, we are optimistic about the future of nano antivirals. The application of antiviral NPs in clinical scenarios such as medical laboratory technology, blood purification and medical protection is expected. As the application of antiviral NPs in the above scenarios continues to develop, the clinical antiviral treatment strategy can be further improved to ‘small molecules as the mainstay and nanoscale as the supplement’. This refinement not only provides a strong support for clinical antiviral treatment, but also offers the experience to promote the clinical transformation of antiviral nanomedicines. We believe that in the future, with multidisciplinary and cross-sector cooperation, nanomedicine-based product development strategies can combat viral infections and improve people's quality of life.

## CRediT authorship contribution statement

**Yicheng Pu:** Writing – review & editing, Writing – original draft, Visualization, Resources, Project administration, Methodology, Formal analysis, Data curation, Conceptualization. **Chuanda Zhu:** Writing – review & editing, Writing – original draft, Formal analysis, Data curation, Conceptualization. **Jun Liao:** Writing – review & editing, Supervision. **Lidong Gong:** Supervision, Funding acquisition. **Yijuan Wu:** Validation, Supervision. **Shunquan Liu:** Supervision. **Hongjun Wang:** Supervision. **Qiang Zhang:** Writing – review & editing, Supervision, Project administration. **Zhiqiang Lin:** Writing – review & editing, Validation, Funding acquisition, Conceptualization.

## Ethics approval and consent to participate

Not applicable for this review.

## Declaration of competing interest

The authors declare the following personal relationships which may be considered as potential competing interests: Hongjun Wang is currently employed by Beijing Tide Pharmaceutical Co., Ltd.
